# Multiple forms of discrimination and relationships with health and wellbeing: findings from national cross-sectional surveys in Aotearoa/New Zealand

**DOI:** 10.1186/s12939-018-0735-y

**Published:** 2018-02-17

**Authors:** Donna Cormack, James Stanley, Ricci Harris

**Affiliations:** 1 Te Rōpū Rangahau Hauora a Eru Pomare, Department of Public Health, University of Otago Wellington, PO Box 7343, Wellington, 6242 New Zealand; 2 Biostatistics Group, Dean’s Department, University of Otago Wellington, Wellington, New Zealand

## Abstract

**Background:**

The complex ways in which experiences of discrimination are patterned in society, including the exposure of communities to multiple overlapping forms of discrimination within social systems of oppression, is increasingly recognised in the health sciences. However, research examining the impacts on health and contribution to racial/ethnic health inequities remains limited. This study aims to contribute to the field by exploring the prevalence and patterning of experience of multiple forms of discrimination in Aotearoa/New Zealand, and associations with health and wellbeing.

**Methods:**

The study’s conceptual approach is informed by Kaupapa Māori theory, Ecosocial theory, Critical Race Theory and intersectionality. Data are from the 2008, 2010 and 2012 General Social Surveys (GSS), biennial nationally-representative surveys in Aotearoa/New Zealand. We examined patterning of forms of discrimination in the last 12 months and frequency of experiencing multiple forms of discrimination. We also looked at associations between experience of multiple discrimination and self-rated health, mental health (using SF12), and life satisfaction using logistic regression. We used random effects meta-analysis to produce pooled estimates drawing from all three survey instances.

**Results:**

Māori, and people from Pacific and Asian ethnic groups, reported much higher prevalence of racial discrimination, were more likely to have any experience of discrimination, and were also more likely to experience multiple forms of discrimination, in the last year relative to respondents in the European/Other category. Discrimination was associated with poorer self-rated health, poorer mental health, and greater life dissatisfaction in unadjusted and adjusted estimates. Negative health impacts increased as the number of forms of discrimination experienced increased.

**Conclusions:**

Discrimination impacts negatively on the health of indigenous peoples and those from minoritised ethnic groups in Aotearoa/New Zealand through higher exposure to racial discrimination, other forms of discrimination, and a greater likelihood of experiencing multiple forms of discrimination. This supports the need for research and interventions that more fully account for the multiple and interlocking ways in which discrimination impacts on health in racialised social hierarchies to maintain systems of privilege and oppression.

**Electronic supplementary material:**

The online version of this article (10.1186/s12939-018-0735-y) contains supplementary material, which is available to authorized users.

## Background

The structural determinants of racial/ethnic health inequities have been recognised for many years within communities and by scholars such as W.E.B. du Bois [1–3]. In recent decades, the social drivers of health and health inequity have received broader acknowledgment in the health sciences. This includes increased research attention on racism as a health determinant, with compelling international evidence of its myriad effects on the health of individuals, communities, and nations over time and across place [4–6]. The relatively rapid development in studies assessing health impacts of discrimination has contributed significantly to understandings of pathways by which racism, as one form of discrimination, impacts negatively on the lives of those marginalised within oppressive racialised social hierarchies, while simultaneously entrenching advantages for those who occupy privileged social positions [4, 6]. Less attention has focused on the ways in which experiences of other discriminations alongside racism may impact health and wellbeing and (re)produce health inequities [7–10], although key scholars have indicated the importance of research that examines “... the cumulative embodiment of multiple types of discrimination, deprivation, and other harmful exposures” [4], (p. 942). Our study aims to contribute to this field by exploring the health impacts of racism and other forms of discrimination in Aotearoa/New Zealand.

### Conceptual approach

This study is influenced by principles drawn from Kaupapa Māori theory [11, 12], Ecosocial theory [4, 9, 13], Critical Race Theory [14, 15], and Intersectionality [16–18]. While these theoretical positions have distinct histories and features, they share a focus on connections between peoples and their broader environments and contexts, recognise the complex realities people have within society [9, 11, 14, 15, 17], and are explicit in their commitment to engaging with critical social issues, including oppression and privilege [11, 14]. Drawing on Windsor et al. [19], oppression is understood “… as a multidimensional and complex hegemonic system developed from social beliefs in group superiority that justify privilege” (p. 22). Discrimination flows from this ‘system’ and is conceptualised in this study in line with the work of Krieger [4] as “… a socially structured and sanctioned phenomenon, justified by ideology and expressed in interactions among and between individuals and institutions, that maintains privileges for members of dominant groups at the cost of deprivation for others” (p. 650). Discrimination is behavioural, encompassing actions and practices with unfair negative impacts for some social groups and advantages for others [4, 20]. The conceptual framework for this study acknowledges the need to understand the ways in which different types of discriminations operate together, as people are often exposed to multiple and intertwined forms of discrimination within systems of oppression and privilege, related to their perceived group memberships or social position in the broader structure over the lifecourse and inter-generationally [4, 7, 17, 21]. Within this conceptual approach, and aligned with understandings of racialisation [22], the research gaze is on interrogating processes by which social group memberships and identities become significant in relation to a particular health outcome [22] through the different, and sometimes simultaneous, forms of discrimination and privilege people experience within an oppressive system, rather than narrowly focusing on the social identities themselves. In this paper, multiple discrimination is used to refer to the experience of multiple forms of discrimination on the basis of more than one grounds, whether this is co-occurring or experienced at different times.

In colonial societies including Aotearoa/New Zealand, racism is a fundamental dimension of the ‘system’ of oppression that shapes the lives, opportunities, and exposures of all people in ways that create and sustain racialised hierarchies of privilege and disadvantage [18]. Racism represents an enduring social phenomenon encompassing racialised beliefs, ideologies, structures, and discriminatory practices [22, 23]. In line with our theoretical approach, this current study recognises the primacy of racialisation in colonial contexts such as Aotearoa/New Zealand, with impacts for indigenous peoples, as well as colonial and migrant populations [14, 18]. Colonisation of Aotearoa/New Zealand by England in the nineteenth Century provides a starting point for understanding both the historical and contemporary context of racialised social relations between Māori as the indigenous peoples of Aotearoa/New Zealand and the New Zealand European (also referred to as Pākehā) colonial settler population. The centrality of racism to colonialism in Aotearoa/New Zealand is reflected in racially-structured access to social, political and economic resources that manifests as privileged social outcomes for New Zealand European/Pākehā, and in stark racialised inequities in health status between New Zealand European/Pākehā and Māori (who make up 15% of the population) [24–26]. Inequities are also evident for other ethnic groups in Aotearoa/New Zealand, including Pacific peoples, who represent 7% of the population. The pattern is less consistent for Asian ethnic groups (12% of the population), although the aggregation of a number of different ethnic groups within the broad categories of Pacific and Asian in official statistics may be masking some inequities [24–27].

### Health impacts of exposure to multiple forms of discrimination

Experiences of multiple forms of discrimination, and pathways between multiple discriminations and health, have been variously conceptualised in the literature. This includes research that investigates experiences of those with “dual minority status” or multiple stigmatised identities (e.g., [28–30]), also conceptualised in terms of “double jeopardy” or “multiple jeopardy” [31]. It has been posited that experiencing multiple stigmatised identities might result in “unique stressors” [28] (p. 3) through experience of multiple forms of discrimination with negative health effects [32]. In addition, exposure to multiple discriminations may impact on use and experience of health services [7], with potential future health effects.

The body of empirical quantitative research examining relationships between experience of multiple discriminations and health is relatively small. Literature specifically focused on the health impacts of racial discrimination alongside other forms of discrimination is concentrated within the United States, with only a few studies from other countries (e.g., Australia [7], Brazil [33], and Canada [34]). A number of these studies have been carried out with specific population groups, such as those living with HIV/AIDS (e.g., [34–37]) or low-income populations (e.g., [38, 39]). Mental health outcomes are the most commonly examined (e.g., [28, 29, 32–34, 37–46, 48–52]), although physical health/self-rated health (e.g., [29, 36, 44, 46]), health behaviours (e.g., [35, 40, 45, 53]), wellbeing (e.g., [32, 40, 47]) and healthcare outcomes [7] have also been assessed. Direct comparisons are difficult due to the variability of the measurement of discrimination across the studies. Studies have found associations between multiple discrimination and negative health outcomes (e.g., [10, 29, 32, 33, 36, 39–41, 43–45, 48], although some findings are mixed (e.g., [39]).

### The current study

International evidence suggests minoritised ethnic groups report more experiences of multiple discrimination [51, 54]. In Aotearoa/New Zealand this is indicated by an increase over time in the number of claims to the Human Rights Commission reporting multiple grounds of discrimination [55]. The current study aims to examine how discrimination experienced on the basis of multiple grounds (multiple discrimination) operates to impact health and contribute to health inequities between ethnic groups in Aotearoa/New Zealand. This is of particular interest regarding indigenous health in Aotearoa/New Zealand, in light of our colonial context and ongoing health inequities. We hypothesised that people from indigenous and other minoritised ethnic groups in Aotearoa/New Zealand (i.e. those from non-European ethnic groups) would experience more forms of discrimination, and that this would be associated with negative impacts on health and wellbeing. In this paper, we present findings of analyses examining the effects of experience of racism and other forms of discrimination on adult health and wellbeing in Aotearoa/New Zealand. Specifically, this paper reports on the patterning of different forms of discrimination in the last twelve months (e.g., racism, sexism) by demographic characteristics; the prevalence of multiple forms of discrimination by ethnicity; impacts of experiencing multiple forms of discrimination on health and wellbeing; and, how different discriminations may act together to impact health and wellbeing.

## Methods

### Data sources

Data for this study were from three instances of the General Social Survey (GSS), a nationally-representative survey undertaken every 2 years (biennially) by Statistics New Zealand [56]. Multiple survey instances were used to increase the overall precision of estimates of association with health outcomes, with results from individual survey analyses pooled using meta-analysis (see Statistical Analysis section). The survey is carried out with adults aged 15 years and over, and asks about a range of social and cultural factors, including culture and identity, human rights, and health [56]. Sampling is undertaken using a three-stage process (including area based sampling as detailed elsewhere [56]), with data collected from participants via CAPI interviews. The first GSS was carried out in 2008. At the time of this project, data were available from the 2008 (*n* = 8721), 2010 (*n* = 8550), and 2012 (*n* = 8462) surveys, with response rates of 83%, 81%, and 78% respectively [56–58]. Survey content was consistent across the three survey instances for the measures of interest in this study. Data were available as Confidentialised Unit Record Files (CURF) from Statistics New Zealand, with ethics approval from the University of Otago Human Ethics Committee (Reference D14/308).

### Measures

#### Discrimination

Discrimination is assessed in the GSS using a stepped question that initially asks: “*In the last 12 months, have you been treated unfairly or had something nasty done to you because of the group you belong to or seem to belong to?*”. Where the response was “yes”, respondents were then asked about frequency (i.e., once, two or three times, or more than three times), followed by a question about the settings where the discrimination occurred (e.g. at home, at work or while working, using transport of any kind). Finally, respondents were asked to indicate what they thought was the grounds for the discrimination for each identified setting, with the ability to select multiple reasons for any setting (full questionnaires for each survey instance are available online [59]). Response options for bases of discrimination align with human rights legislation in Aotearoa/New Zealand, and include: my skin colour; my nationality, race or ethnic group; the language I speak; the way I dress or my appearance; my gender (male or female); my age; a disability or health issue I have; my marital status (whether or not you are married or living with someone); my family status (whether or not you have children); my sexual orientation (lesbian/gay/straight/bisexual/transgender); what I do for a job; my religious beliefs; my political position; other; don’t know; and, refused. In our analysis, we examine only the reason given for any discrimination reported in the last year, but not the setting where it occurred or a measure of overall frequency of discrimination. In line with our conceptual approach and the human rights context in Aotearoa/New Zealand, we grouped discrimination on the basis of “skin colour”, “race, ethnicity and nationality”, and the “language” spoken to form a ‘racial discrimination’ category. The other bases for discrimination were considered separately. The GSS residual category “other reason given” was treated as its own category. To consider multiple discrimination, discrimination variables were grouped into six categories for analysis: ‘Racism only’ (R1), where respondents reported racism as the only basis for discrimination; ‘Racism plus one’ (R2), where respondents reported racism and one other type of discrimination; ‘Racism 3 or more’ (R3+), where respondents reported racism and two or more other types of discrimination; ‘No R [acism], One other’ (D1), where respondents reported one basis of discrimination, but not racism; ‘No R [acism], 2 others’ (D2), where respondents reported 2 bases of discrimination, but not racism; and, ‘No R [acism], 3 others’ (D3+), where respondents reported 3 or more bases of discrimination, but not racism. In our decisions about categorisation, we decided to group multiple discriminations so that it was possible to identify racial discrimination separately from other types of discrimination, in line with our conceptual approach.

#### Health and wellbeing measures

The GSS includes several health and wellbeing variables. For this study, we selected one measure each for general health, mental health, and wellbeing domains [59]. The self-rated general health question was used to assess general health, with response options dichotomised as poor/fair versus good/very good/excellent, in line with previous studies of racism and general self-rated health [60]. The SF-12 mental health component summary score was analysed as a continuous variable. Finally, a question on overall life satisfaction was used as a measure of wellbeing. This question asked respondents “*How do you feel about your life as a whole right now?*”, with possible response options of: ‘very satisfied’; ‘satisfied’; ‘no feeling either way’; ‘dissatisfied’; and, ‘very dissatisfied’. Responses were grouped into ‘dissatisfied/very dissatisfied’ versus all else, providing a measure of current life dissatisfaction.

#### Other measures

The GSS collects self-identified ethnicity from all respondents using the standard question from the New Zealand Population Census. Although individuals may identify with multiple ethnic groups, the CURF pre-aggregates these responses into broad ethnic groupings of: Māori, Asian ethnic groups, Pacific peoples; European; and Other ethnic groups. In the CURF dataset, the final group (European/Other) could not be disaggregated for the 2008 GSS; based on estimates from the 2010/2012 data, around 4% of individuals in this composite group identified with a non-European ethnicity. In the analysis, Māori, Asian and Pacific ethnic groupings were categorised using ‘total ethnicity’ approach for descriptive analyses (with individuals counted in each of the broad ethnic categories they identified with). As the comparator group, the ‘European/Other’ category included those participants who only identified with a ‘European’ or an ‘Other’ ethnic group. In modelling, participants who identified with more than one ethnic grouping were counted in only one ethnic category, based on a pre-determined hierarchy (Māori, Pacific, Asian, All else) in line with standard processes used in the health sector in Aotearoa/New Zealand [61].

Other variables included in analysis were gender and age as confounders, and two measures of socioeconomic position conceptualised as mediators [59]. Gender was analysed as male/female, and age analysed as age groups (15–24, 25–34, 35–44, 45–64, 65–74, 75+ years). The New Zealand Index of Deprivation (NZDep) 2006 was included as an area-based measure of relative deprivation [62]. NZDep2006 uses nine variables from the 2006 Population Census to calculate relative deprivation scores from 1 to 10 for small areas (referred to as ‘meshblocks’). In brief, the variables include: receiving a benefit; school qualification; access to a telephone; access to a car; equivalised household income; household type (sole-parent); number of people in household; living in own home; and, employment status [62]. For analysis, NZDep2006 was grouped from quintiles 1 (least deprived) to 5 (most deprived). Educational qualification was included as a measure of individual socioeconomic position, and categorised as ‘no secondary qualification’ vs. ‘secondary qualification or higher’.

### Statistical analysis

The complex survey design was accounted for in all analyses using jackknife based weights provided by Statistics NZ, which account for both inverse sampling weighting of respondents to produce nationally representative estimates and components to handle clustering of responses introduced by the area-based sampling [56–58]. Descriptive statistics (e.g. means, frequencies and prevalences) were produced as appropriate for sociodemographic, exposure, and outcome variables. Prevalence of discrimination by basis of discrimination (e.g. racism, age) was analysed separately for each survey instance (2008, 2010, and 2012).

Regression models were constructed to examine the association between exposure to discrimination and each outcome variable, with separate analyses for each of the three survey instances. Models are presented with sequential adjustment across three models: firstly, unadjusted estimates; then adjusted for sociodemographic confounders (age and gender); and then further adjusted for socioeconomic confounders/mediators (NZDep and educational qualification).

Logistic regression models were used for the two categorical outcomes (self-rated health and life satisfaction) using PROC SURVEYLOGISTIC in SAS 9.2 (SAS Institute, North Carolina); linear regression was used for the continuous outcome (SF-12 mental health score) using the svy prefix with the reg command in Stata 12 (Statacorp, Texas). For each outcome variable, the resulting parameter estimates (log odds ratios for logistic regression; slope coefficients for linear regression) from the models for each survey instance were then combined using random-effects meta-analysis to produce pooled estimates drawing from all three survey instances (2008, 2010, 2012). Variant models were run to test for trend effects across increasing levels of discrimination: this was done by including two ordinal variables in the model (one for the R1/R2/R3+ levels, and another for the D1/D2/D3+ levels). These estimates from the three separate GSS instances were again passed through a meta-analysis to produce a pooled estimate.

## Results

Table 1 summarises participant characteristics and the prevalence of health and wellbeing outcomes and exposure to multiple discriminations for each survey instance (GSS 2008, 2010, 2012). The patterning of demographic characteristics, and exposure and outcome variables is similar across the three time periods (Table 1).

**Table 1:** GSS participant characteristics by survey instance (2008, 2010, 2012)

	Survey instance (GSS Year)					
	2008		2010		2012	
	*n*	%	*n*	%	*n*	%
*Total (n)*	8721	100.0	8550	100.0	8462	100.0
*Ethnicity*						
Māori	972	11.1	947	11.1	1114	13.2
Pacific	385	4.4	289	3.4	421	5.0
Asian	540	6.2	571	6.7	647	7.6
European/Other	6865	78.7	6779	79.3	6348	75.0
*Age group*						
15–24	958	11.0	912	10.7	935	11.0
25–34	1277	14.6	1146	13.4	1217	14.4
35–44	1690	19.4	1652	19.3	1467	17.3
45–54	1547	17.7	1502	17.6	1519	18.0
55–64	1317	15.1	1359	15.9	1379	16.3
65–74	1021	11.7	1033	12.1	1074	12.7
75+	911	10.4	946	11.1	871	10.3
*Gender*						
F	4800	55.0	4773	55.8	4749	56.1
M	3921	45.0	3777	44.2	3713	43.9
*Education*						
No secondary qualification	2296	26.3	2077	24.3	2041	24.1
At least secondary qualification	6405	73.4	6459	75.5	6412	75.8
*NZ Dep Quintiles*						
1	1561	17.9	1518	17.8	1418	16.8
2	1830	21.0	1784	20.9	1494	17.7
3	1890	21.7	1874	21.9	1534	18.1
4	1924	22.1	1898	22.2	2094	24.7
5	1488	17.1	1449	16.9	1922	22.7
*Self-rated health*						
Poor/fair	1286	14.7	1287	15.1	1344	15.9
Excellent/very good/good	7435	85.3	7260	84.9	7112	84.0
*Life satisfaction*						
[Very] dissatisfied	660	7.6	617	7.2	599	7.1
[Very] satisfied/ No feeling either way	8050	92.3	7926	92.7	7856	92.8
*SF-12 Mental Health*						
Mean (95% CI)	8655	52.4 (52.2, 52.7)	8496	50.0 (49.7, 50.3)	8405	50.2 (49.9, 50.5)
*Experience of discrimination*						
No discrimination	7904	90.6	7694	90.0	7633	90.2
Racism only (R1)	241	2.8	232	2.7	253	3.0
Racism plus one (R2)	109	1.2	102	1.2	82	1.0
Racism 3+ (R3+)	97	1.1	108	1.3	113	1.3
No R, one other (D1)	242	2.8	282	3.3	262	3.1
No R, 2 others (D2)	78	0.9	75	0.9	69	0.8
No R, 3 + (D3+)	50	0.6	57	0.7	50	0.6

### Reported basis for discrimination

Table 2 shows the patterning of racial and other forms of discrimination in the last 12 months, using data from the 2012 GSS (see Additional files 1: Table S1 and Table S2 for 2008 and 2010 results respectively). Racial discrimination was the most commonly reported basis of discrimination in the last year, with Māori, and Pacific and Asian ethnic groupings reporting much higher prevalence (10.3%, 9.0% and 14.1% respectively) of racial discrimination compared with the European/Other grouping (3.1%). Māori also reported higher experience of discrimination for non-racial reasons relative to the European/Other grouping, for most other bases except for marital status and religion (Table 2). Aside from gender discrimination (and potentially family status), patterning of reasons for discrimination were relatively similar between males and females. For most forms of discrimination, reporting seemed to decrease with age. Patterns by education were not consistent across the forms of discrimination, although there appeared to be some evidence for a deprivation gradient in reporting discrimination for at least some bases of discrimination, with greater discrimination with increasing deprivation for several types of discrimination including racial discrimination. In general, patterns were similar in the 2008 and 2010 GSS instances (see Additional files 1: Table S1 and S2).

**Table 2:** Patterning of racial and other forms of discrimination in last 12 months, General Social Survey 2012

	Racism	Dress/appearance	Gender	Age	Disability	Marital status	Family	Sexual orientation	Occupation	Religion	Political	Other	
	% (95% CI)	% (95% CI)	% (95% CI)	% (95% CI)	% (95% CI)	% (95% CI)	% (95% CI)	% (95% CI)	% (95% CI)	% (95% CI)	% (95% CI)	% (95% CI)	n
*Ethnic grouping*													
Māori	10.3 (8.1, 12.4)	4.3 (2.8, 5.7)	2.8 (1.5, 4.1)	3.2 (1.9, 4.4)	1.9 (0.9, 2.9)	0.8 (0.3, 1.2)	1.2 (0.6, 1.7)	0.6 (0.0, 1.2)	1.9 (0.7, 3.0)	1.3 (0.3, 2.3)	1.1 (0.4, 1.9)	4.2 (2.4, 6.0)	1114
Pacific	9.0 (5.8, 12.1)	2.5 (0.9, 4.0)	1.8 (0.5, 3.2)	1.6 (0.3, 2.8)	1.2 (0.0, 2.5)	0.1 (0.0, 0.3)	0.1 (0.0, 0.2)	0.7 (0.0, 1.6)	1.9 (0.3, 3.5)	1.7 (0.3, 3.0)	0.2 (0.0, 0.4)	1.7 (0.4, 2.9)	421
Asian	14.1 (10.7, 17.6)	2.8 (0.0, 5.7)	1.4 (0.5, 2.4)	2.3 (0.1, 4.6)	0.2 (0.0, 0.4)	1.3 (0.0, 3.5)	0.3 (0.0, 0.7)	–	2.1 (0.0, 4.4)	2.3 (0.0, 4.7)	0.2 (0.0, 0.5)	1.4 (0.4, 2.4)	647
Euro/Other	3.1 (2.6, 3.6)	1.2 (0.9, 1.5)	1.1 (0.8, 1.4)	1.1 (0.8, 1.4)	1.0 (0.7, 1.2)	0.5 (0.3, 0.7)	0.2 (0.1, 0.4)	0.4 (0.2, 0.6)	0.9 (0.6, 1.3)	0.8 (0.5, 1.0)	0.3 (0.1, 0.5)	1.8 (1.3, 2.2)	6348
*Gender*													
Female	5.1 (4.4, 5.8)	1.9 (1.5, 2.4)	2.0 (1.5, 2.5)	1.8 (1.4, 2.3)	1.1 (0.8, 1.4)	0.6 (0.4, 0.8)	0.5 (0.3, 0.7)	0.4 (0.2, 0.7)	1.0 (0.6, 1.4)	1.0 (0.6, 1.4)	0.3 (0.2, 0.4)	2.0 (1.5, 2.6)	4749
Male	5.8 (4.7, 6.9)	1.7 (1.0, 2.4)	0.7 (0.3, 1.0)	1.1 (0.5, 1.7)	0.8 (0.5, 1.1)	0.6 (0.0, 1.1)	0.2 (0.0, 0.3)	0.3 (0.1, 0.5)	1.4 (0.8, 2.1)	1.0 (0.4, 1.7)	0.5 (0.2, 0.8)	2.0 (1.4, 2.6)	3713
*Age group*													
15–24	6.3 (4.1, 8.5)	4.7 (2.6, 6.7)	1.5 (0.7, 2.3)	2.8 (1.1, 4.6)	1.0 (0.2, 1.7)	1.1 (0.0, 2.6)	0.4 (0.1, 0.7)	0.6 (0.1, 1.1)	1.7 (0.2, 3.2)	2.0 (0.2, 3.8)	0.2 (0.0, 0.5)	2.2 (1.1, 3.4)	935
25–34	7.1 (5.4, 8.9)	2.0 (1.1, 2.9)	1.7 (0.9, 2.6)	1.4 (0.7, 2.1)	1.2 (0.5, 1.9)	0.9 (0.4, 1.5)	0.6 (0.1, 1.0)	0.5 (0.0, 1.0)	1.1 (0.4, 1.8)	1.0 (0.3, 1.7)	0.7 (0.1, 1.3)	2.4 (1.5, 3.4)	1217
35–44	6.6 (4.8, 8.4)	1.5 (0.8, 2.2)	1.9 (0.9, 2.8)	0.6 (0.2, 1.0)	1.2 (0.6, 1.8)	0.7 (0.3, 1.2)	0.7 (0.2, 1.1)	0.5 (0.1, 0.9)	0.9 (0.4, 1.5)	0.8 (0.3, 1.3)	0.2 (0.0, 0.5)	2.6 (1.4, 3.8)	1467
45–54	6.8 (5.1, 8.5)	1.3 (0.7, 1.9)	1.6 (0.9, 2.2)	1.3 (0.5, 2.1)	1.0 (0.6, 1.4)	0.4 (0.1, 0.8)	0.2 (0.0, 0.5)	0.3 (0.0, 0.7)	1.9 (0.9, 2.8)	0.9 (0.4, 1.4)	0.6 (0.2, 1.1)	2.6 (1.3, 3.9)	1519
55–64	3.3 (2.3, 4.4)	0.8 (0.3, 1.3)	1.1 (0.5, 1.8)	1.8 (1.0, 2.5)	1.1 (0.6, 1.6)	0.1 (0.0, 0.1)	0.1 (0.0, 0.2)	0.3 (0.0, 0.7)	1.4 (0.6, 2.2)	1.2 (0.4, 1.9)	0.3 (0.0, 0.9)	1.8 (0.9, 2.8)	1379
65–74	3.0 (1.4, 4.6)	0.3 (0.0, 0.8)	0.3 (0.0, 0.5)	1.0 (0.5, 1.6)	0.2 (0.0, 0.4)	0.2 (0.0, 0.4)	–	–	0.3 (0.0, 0.7)	0.2 (0.0, 0.5)	0.2 (0.0, 0.5)	0.4 (0.1, 0.7)	1074
75+	0.7 (0.1, 1.3)	–	0.3 (0.0, 0.8)	1.0 (0.3, 1.8)	0.1 (0.0, 0.2)	–	–	–	–	0.3 (0.0, 0.7)	0.2 (0.0, 0.6)	0.2 (0.0, 0.6)	871
*Education*													
No secondary qualification	4.6 (3.3, 5.9)	1.9 (1.2, 2.6)	1.2 (0.4, 2.0)	0.9 (0.5, 1.4)	1.3 (0.6, 1.9)	0.5 (0.1, 0.9)	0.3 (0.1, 0.5)	0.3 (0.0, 0.6)	0.8 (0.4, 1.3)	0.8 (0.2, 1.4)	0.1 (0.0, 0.3)	2.0 (1.1, 2.8)	2041
Secondary qualification	5.7 (4.9, 6.4)	1.8 (1.2, 2.3)	1.4 (1.1, 1.7)	1.6 (1.2, 2.1)	0.9 (0.6, 1.1)	0.6 (0.3, 0.9)	0.3 (0.2, 0.4)	0.4 (0.2, 0.6)	1.3 (0.9, 1.7)	1.1 (0.7, 1.5)	0.4 (0.3, 0.6)	2.0 (1.6, 2.5)	6412
*Quintile*													
Quintile 1	3.6 (2.4, 4.8)	0.7 (0.2, 1.2)	1.2 (0.5, 1.8)	0.8 (0.3, 1.2)	0.3 (0.0, 0.5)	0.2 (0.0, 0.3)	0.2 (0.0, 0.5)	0.2 (0.0, 0.5)	0.7 (0.2, 1.1)	0.2 (0.0, 0.4)	0.1 (0.0, 0.2)	1.3 (0.6, 2.0)	1418
Quintile 2	4.0 (2.7, 5.3)	0.7 (0.2, 1.1)	0.8 (0.4, 1.3)	1.0 (0.5, 1.6)	0.7 (0.3, 1.1)	0.6 (0.2, 0.9)	0.3 (0.1, 0.6)	0.3 (0.0, 0.6)	1.5 (0.7, 2.2)	1.1 (0.3, 1.9)	0.6 (0.0, 1.1)	2.1 (0.9, 3.3)	1494
Quintile 3	5.3 (4.1, 6.5)	1.8 (1.0, 2.6)	1.3 (0.7, 1.9)	1.5 (0.8, 2.2)	0.8 (0.3, 1.2)	0.4 (0.1, 0.7)	0.2 (0.0, 0.5)	0.2 (0.0, 0.5)	1.3 (0.5, 2.1)	1.3 (0.6, 2.0)	0.3 (0.0, 0.5)	2.1 (1.1, 3.1)	1534
Quintile 4	7.2 (5.5, 9.0)	2.9 (1.2, 4.5)	1.6 (0.9, 2.4)	2.7 (1.3, 4.1)	1.3 (0.8, 1.9)	1.3 (0.1, 2.5)	0.4 (0.1, 0.6)	0.7 (0.2, 1.2)	1.7 (0.4, 3.1)	1.7 (0.4, 3.0)	0.6 (0.2, 1.0)	2.3 (1.4, 3.2)	2094
Quintile 5	7.8 (6.2, 9.3)	3.4 (2.2, 4.6)	1.9 (1.2, 2.7)	1.5 (0.9, 2.0)	1.8 (1.1, 2.6)	0.5 (0.2, 0.9)	0.4 (0.1, 0.8)	0.5 (0.1, 0.8)	0.8 (0.2, 1.3)	0.8 (0.3, 1.3)	0.4 (0.1, 0.7)	2.4 (1.7, 3.2)	1922

Table notes: % are weighted, but numbers (n) are unweighted; Euro/Other is an abbreviation for the European/Other category; cells marked as “-” are not reported due to small cell sizes and confidentiality rules

### Single and multiple discriminations

Table 3 shows the relationship between single and multiple experiences of racism and other forms of discrimination by participant characteristics and health and wellbeing outcomes. It presents data for the latest GSS (2012). Other survey years are appended (Additional files 1: Table S3 and S4), although general patterning is similar. Most respondents reported no discrimination in the last 12 months (Table 3). However, Māori, Pacific and Asian ethnic groupings were more likely to report at least one experience of discrimination compared with the European/Other grouping, who had the highest prevalence of no reported discrimination in the last 12 months (92.5%). Māori, Pacific and Asian ethnic groups were also more likely to report experiencing multiple discriminations compared with the European/Other group, although this was difficult to assess in the non-racial discrimination only categories because of small numbers. Experience of multiple forms of discrimination appeared to increase with increasing deprivation, although the differences were not significant. Experience of both single and multiple forms of discrimination tended to be higher among participants who had more negative health outcomes (i.e. among those who had poor or fair self-rated health, general life dissatisfaction and lower mental health scores).

**Table 3:** Discrimination by participant characteristics and health outcomes, General Social Survey 2012

	Total	Racism only (R1)		Racism plus one (R2)		Racism 3+ (R3+)		No R, one other (D1)		No R, 2 others (D2)		No R, 3 + (D3+)		None	
	n	n	% (95% CI)	n	% (95% CI)	n	% (95% CI)	n	% (95% CI)	n	% (95% CI)	n	% (95% CI)	n	% (95% CI)
Total (n)	8462	253	3.1 (2.6, 3.6)	82	0.9 (0.7, 1.2)	113	1.4 (1.1, 1.8)	262	2.9 (2.4, 3.4)	69	0.6 (0.4, 0.8)	50	0.5 (0.4, 0.7)	7633	90.5 (89.5, 91.4)
*Ethnicity*															
Māori	1114	44	3.9 (2.5, 5.4)	27	2.1 (1.3, 3.0)	41	4.2 (2.6, 5.7)	44	4.0 (2.3, 5.7)	16	1.1 (0.4, 1.8)	6	0.3 (0.0, 0.7)	936	84.3 (81.5, 87.1)
Pacific	421	24	4.2 (2.3, 6.1)	6	1.6 (0.0, 3.2)	17	3.1 (1.2, 5.1)	6	1.1 (0.2, 1.9)	4	0.4 (0.0, 0.9)	0	–	364	89.6 (86.4, 92.8)
Asian	647	70	8.5 (6.3, 10.7)	22	3.1 (1.6, 4.7)	10	2.5 (0.3, 4.7)	13	1.5 (0.7, 2.4)	0	–	1	0.1 (0.0, 0.2)	531	84.3 (80.7, 87.8)
European/Other	6348	118	2.0 (1.5, 2.4)	30	0.4 (0.2, 0.5)	52	0.7 (0.5, 1.0)	200	3.1 (2.4, 3.7)	49	0.7 (0.4, 0.9)	43	0.7 (0.4, 0.9)	5856	92.5 (91.7, 93.4)
*Age group*															
15–24	935	25	2.4 (1.3, 3.5)	14	1.5 (0.5, 2.4)	22	2.4 (0.7, 4.1)	39	3.7 (2.2, 5.1)	11	0.7 (0.2, 1.3)	9	0.6 (0.2, 1.0)	815	88.7 (85.6, 91.8)
25–34	1217	41	3.9 (2.6, 5.2)	19	1.7 (0.9, 2.5)	22	1.5 (0.8, 2.3)	35	2.3 (1.4, 3.2)	14	0.9 (0.4, 1.5)	10	0.8 (0.2, 1.3)	1076	88.8 (86.8, 90.9)
35–44	1467	58	3.9 (2.5, 5.3)	13	0.6 (0.2, 0.9)	32	2.1 (1.1, 3.1)	47	3.2 (2.0, 4.5)	9	0.4 (0.1, 0.7)	7	0.4 (0.1, 0.8)	1301	89.3 (87.1, 91.5)
45–54	1519	68	4.5 (3.0, 5.9)	17	1.0 (0.4, 1.5)	20	1.4 (0.7, 2.1)	57	3.6 (2.2, 5.0)	15	0.8 (0.2, 1.3)	14	0.6 (0.2, 1.0)	1328	88.2 (86.0, 90.4)
55–64	1379	35	2.0 (1.1, 2.9)	13	0.7 (0.2, 1.1)	12	0.7 (0.2, 1.2)	54	3.5 (2.4, 4.6)	16	0.9 (0.4, 1.5)	8	0.6 (0.0, 1.2)	1241	91.6 (89.7, 93.5)
65–74	1074	19	2.2 (0.9, 3.5)	6	0.5 (0.0, 1.0)	4	0.3 (0.0, 0.7)	18	1.3 (0.6, 1.9)	4	0.2 (0.0, 0.4)	1	0.1 (0.0, 0.3)	1022	95.5 (93.7, 97.2)
75+	871	7	0.4 (0.1, 0.8)	0	–	1	0.3 (0.0, 0.8)	12	1.0 (0.3, 1.6)	0	–	1	0.2 (0.0, 0.6)	850	98.2 (97.2, 99.1)
*Gender*															
F	4749	125	2.4 (1.9, 2.9)	45	1.0 (0.6, 1.3)	79	1.7 (1.3, 2.1)	154	3.1 (2.5, 3.8)	44	0.8 (0.4, 1.1)	31	0.6 (0.4, 0.8)	4271	90.4 (89.2, 91.7)
M	3713	128	3.7 (2.8, 4.7)	37	0.9 (0.6, 1.3)	34	1.1 (0.6, 1.7)	108	2.7 (2.0, 3.4)	25	0.5 (0.2, 0.7)	19	0.5 (0.2, 0.7)	3362	90.5 (89.1, 92.0)
*Education*															
No secondary qualification	2041	49	2.5 (1.6, 3.4)	21	1.0 (0.5, 1.5)	20	1.1 (0.5, 1.8)	57	2.7 (1.7, 3.6)	11	0.6 (0.2, 1.1)	8	0.5 (0.1, 0.8)	1875	91.6 (89.9, 93.4)
At least secondary qualification	6412	204	3.2 (2.7, 3.8)	61	0.9 (0.7, 1.2)	93	1.5 (1.1, 1.9)	205	3.0 (2.4, 3.5)	58	0.6 (0.4, 0.9)	41	0.5 (0.3, 0.7)	5750	90.2 (89.1, 91.2)
*NZ Dep Quintiles*															
1	1418	27	2.4 (1.3, 3.5)	6	0.4 (0.1, 0.7)	10	0.8 (0.2, 1.5)	29	1.8 (1.0, 2.7)	5	0.4 (0.0, 0.7)	4	0.3 (0.0, 0.5)	1337	94.0 (92.5, 95.5)
2	1494	31	2.3 (1.4, 3.3)	8	0.6 (0.0, 1.2)	16	1.0 (0.5, 1.6)	43	3.2 (1.8, 4.6)	10	0.5 (0.2, 0.9)	7	0.4 (0.0, 0.8)	1379	91.9 (89.9, 93.9)
3	1534	40	3.0 (2.0, 4.0)	11	0.8 (0.3, 1.3)	23	1.5 (0.8, 2.2)	46	3.0 (1.8, 4.1)	16	0.8 (0.4, 1.1)	6	0.4 (0.1, 0.6)	1392	90.6 (89.0, 92.3)
4	2094	76	3.8 (2.7, 4.9)	26	1.3 (0.7, 1.9)	29	2.1 (0.8, 3.3)	77	3.7 (2.5, 4.8)	17	0.7 (0.2, 1.2)	20	1.1 (0.5, 1.7)	1849	87.3 (85.0, 89.7)
5	1922	79	4.0 (3.0, 5.1)	31	1.9 (1.0, 2.9)	35	1.8 (1.1, 2.5)	67	3.0 (2.0, 3.9)	21	0.9 (0.4, 1.4)	13	0.5 (0.2, 0.9)	1676	87.8 (86.0, 89.7)
*Self-rated health*															
Poor/fair	1344	56	3.9 (2.5, 5.3)	15	1.4 (0.5, 2.3)	36	2.9 (1.8, 4.1)	60	4.2 (2.9, 5.4)	24	1.6 (0.8, 2.4)	18	1.2 (0.4, 2.0)	1135	84.8 (82.5, 87.1)
Excellent/very good/good	7112	197	2.9 (2.4, 3.5)	67	0.9 (0.6, 1.1)	77	1.2 (0.8, 1.6)	202	2.7 (2.2, 3.2)	45	0.5 (0.3, 0.7)	32	0.4 (0.3, 0.6)	6492	91.3 (90.4, 92.3)
*Life satisfaction*															
[Very] dissatisfied	599	34	5.2 (3.1, 7.2)	10	1.1 (0.1, 2.1)	21	4.1 (2.0, 6.2)	39	6.0 (3.5, 8.5)	12	2.1 (0.7, 3.5)	12	1.4 (0.5, 2.3)	471	80.1 (76.4, 83.8)
[Very] satisfied/ No feeling either way	7856	219	2.9 (2.4, 3.5)	72	0.9 (0.7, 1.2)	92	1.3 (0.9, 1.6)	223	2.7 (2.2, 3.2)	57	0.6 (0.4, 0.7)	38	0.5 (0.3, 0.6)	7155	91.1 (90.2, 92.1)
*SF-12 Mental Health*															
Mean (95% CI)	8405	47.3	(45.5, 49.0)	45.2	(42.5, 47.9)	41.8	(39.5, 44.1)	46.7	(45.0, 48.5)	41.5	(37.3, 45.7)	37.2	(32.3, 42.1)	50.7	(50.4, 51.0)

Table notes: % are weighted, but numbers (n) are unweighted

### Multiple discrimination and health

Experience of discrimination was associated with worse health for all outcomes assessed, in both unadjusted (Table 4; Additional files 2, 3, 4: Figures S1, S2, S3) and fully-adjusted estimates (Table 4; Additional files 5, 6, 7: Figures S4, S5, S6).

**Table 4:** Associations between experience of multiple discriminations and health (odds ratio and mean difference)

	Unadjusted		Adjusted	
Fair/poor self-rated health	OR	95% CI	OR	95% CI
* Racism only (R1)*	1.41	1.06, 1.89	1.63	1.21, 2.21
* Racism plus one (R2)*	1.94	1.34, 2.79	2.15	1.47, 3.19
* Racism 3+ (R3)*	2.04	1.42, 2.90	2.39	1.57, 3.64
* No R, one other (D1)*	1.78	1.28, 2.48	2.10	1.45, 3.04
* No R, two others (D2)*	3.01	2.07, 4.36	3.89	2.60, 5.83
* No R, 3+ (D3)*	2.67	1.72, 4.14	3.33	2.17, 5.09
Dissatisfied/very dissatisfied	OR	95% CI	OR	95% CI
* Racism only (R1)*	1.79	1.35, 2.36	1.94	1.44, 2.61
* Racism plus one (R2)*	2.21	1.37, 3.58	2.03	1.24, 3.32
* Racism 3+ (R3)*	3.02	2.12, 4.31	2.74	1.89, 3.96
* No R, one other (D1)*	2.66	2.07, 3.42	2.56	1.97, 3.33
* No R, two others (D2)*	3.78	2.23, 6.39	3.87	2.18, 6.89
* No R, 3+ (D3)*	4.18	2.66, 6.59	4.14	2.61, 6.57
Mental health	Mean difference	95% CI	Mean difference	95% CI
* Racism only (R1)*	−3.10	−4.35, −1.86	−3.16	−4.30, −2.02
* Racism plus one (R2)*	−5.30	−7.04, −3.57	−4.94	−6.66, −3.21
* Racism 3+ (R3)*	−6.55	−9.04, −4.05	−6.07	−8.50, −3.65
* No R, one other (D1)*	−4.23	−5.25, −3.21	−4.01	−5.00, −3.02
* No R, two others (D2)*	−6.36	−11.15, −1.58	−5.93	−10.38, −1.49
* No R, 3+ (D3)*	−11.76	− 14.56, −8.95	−11.33	− 14.02, −8.63

The results below concentrate on reporting the fully-adjusted estimates. For self-rated health, experience of racism alone (R1) increased the risk of poor/fair health (adjusted OR = 1.63; 95% CI 1.21, 2.21). This effect was stronger among those reporting racism and one other form of discrimination (R2) (adjusted OR = 2.15; 95% CI 1.47, 3.19) or racism and at least two other forms of discrimination (R3+) (adjusted OR = 2.39; 95% CI 1.57, 3.64) (Table 4, Additional file 5: Figure S4). A test-for-trend across these three levels indicated increased odds of poor/fair health with increasing levels of discrimination (adjusted OR per level = 1.39, 95% CI 1.27, 1.52, *p* < 0.001). A similar pattern was seen amongst those experiencing other forms of discrimination in the absence of racism (for one other form (D1) (adjusted OR = 2.10; 95% CI 1.45, 3.04), two non-racial forms (D2) (adjusted OR = 3.89, 95% CI 2.60, 5.83), and three or more non-racial discriminations (D3+) (adjusted OR = 3.33, 95% CI 2.17, 5.09)). The test-for-trend again indicated increased odds of poor/fair health with increasing levels of discrimination (adjusted OR per level = 1.75, 95% CI 1.58, 1.94, p < 0.001).

For self-rated health, the impact of non-racial discrimination (single and multiple forms) appeared to be stronger than for racial discrimination and multiple discrimination involving racism. However, formal comparison of the difference between these sets of groups (e.g. two forms including racism compared to two forms excluding racism) were not statistically significant (confidence intervals for pairwise contrasts between these groups all included null OR of 1).

The pattern was similar for the life satisfaction measure, with higher odds of dissatisfaction amongst those with exposure to racial and other discriminations (Table 4, Additional file 6: Figure S5). This included a gradient of increased risk with increased experience of multiple discriminations. The odds of reporting dissatisfaction were 1.94 times higher (adjusted OR, 95% CI 1.44, 2.61) among those experiencing racial discrimination only (R1), 2.04 times higher (adjusted OR, 95% CI 1.24, 3.32) among those experiencing racial discrimination and one other form (R2), and 2.74 times higher (adjusted OR, 95% CI 1.89, 3.96) for those experiencing racial discrimination and at least two other forms of discrimination (R3+), which was again supported by a test-for-trend examining increase in odds for each additional type of discrimination (adjusted OR per level = 1.44, 95% CI 1.30, 1.60, *p* < 0.001). As with self-rated health, the point estimates for associations of non-racial discrimination (alone or in combination with other discriminations) tended to be higher than those for racial discrimination (alone or in combination with other discriminations) and the test for trend again indicated increased odds of dissatisfaction with additional types of discrimination (adjusted OR per level = 1.89, 95% CI 1.67, 2.14, *p* < 0.001).

Mean scores for the SF12 Mental Health scale were lower for those exposed to discrimination (Table 4, Additional file 7: Figure S6), indicating poorer mental health, with magnitudes ranging from a mean difference of 3 to 4 points lower (relative to those with no exposure to discrimination) for those with a single form of racial or other discrimination (racism alone (R1), adjusted mean difference = − 3.16, 95% CI -4.30, − 2.02; one non-racial form alone (D1), adjusted mean difference = − 4.01, 95% CI -5.00, − 3.02). Again, there was reasonable evidence for a gradient for poorer mental health status with multiple discriminations, both for the group experiencing multiple forms including racism (adjusted mean difference per extra level = − 2.07, 95% CI -2.63, − 1.51) and amongst those experiencing multiple forms of discrimination without racism (adjusted mean difference per extra level = − 3.65, 95% CI -4.29, − 3.02).

## Discussion

Our study aimed to better understand how exposure to multiple forms of discrimination is associated with health in Aotearoa/New Zealand. In line with our hypothesis and conceptualisation of multiple oppressions within colonial contexts, we found indigenous and minoritised ethnic groups (i.e. Māori and people from Asian and Pacific ethnic groups) were more likely to report substantially higher prevalence of racial discrimination. Māori were also more likely to report higher prevalence for each of a number of other forms of discrimination, including discrimination on grounds of age, gender or appearance. In addition, Māori, Pacific peoples and those from Asian ethnic groups, were more likely to experience at least one form of discrimination, and to experience other forms of discrimination in addition to racial discrimination, in the last 12 months. This aligns with international literature that shows minoritised ethnic groups report significantly more exposure to multiple forms of discrimination (e.g. [44, 54, 63]).

The higher prevalence of racial discrimination for Māori, Pacific peoples and those from Asian ethnic groups, has been documented previously in Aotearoa/New Zealand [60, 64, 65]. However, there is little data on the patterning of other forms of discrimination, such as sexism or ageism [64, 65]. In terms of multiple discrimination in our study, there was some evidence to suggest that likelihood of discrimination in the last year increased with socioeconomic deprivation. This supports a pathway by which socioeconomic inequities in health may also be influenced in part by experiences of multiple forms of discrimination associated with living in areas of higher deprivation. Socioeconomic inequities in Aotearoa/New Zealand are heavily stratified by ethnicity, with NZ European/Pākehā populations having privileged access to higher socioeconomic position and lower deprivation [25]. Our findings suggest privilege may also operate for NZ European/Pākehā through lower exposure to any discrimination and less multiple discrimination. Our understanding of relationships between socioeconomic position and multiple discrimination could be expanded by further research that captures other aspects of socioeconomic position that may be impacting on these associations.

As hypothesised, we found that experiencing multiple forms of discrimination in the past year was associated with negative impacts on health and wellbeing, after controlling for age, gender, educational qualification and area-based deprivation. This pattern was apparent for multiple discrimination involving both racially and non-racially based discrimination, with the impact appearing to increase with the number of different forms of discrimination experienced, suggesting a dose-response relationship. Other studies internationally have also found increasing negative health impacts with increasing report of multiple forms of discrimination (e.g., [7, 10, 39, 40, 45]). Bucchianeri et al. [40], for example found that likelihood of reporting negative substance use, self-harm and emotional wellbeing outcomes increased with increasing accumulation of types of discrimination for adolescents. The point estimates in our study were somewhat higher for experiences of discrimination that were not attributed as racial, which was not entirely expected, although this pattern has been reported elsewhere [39, 66]. The strength of association between different forms of discrimination and health is not entirely consistent in the literature [42, 67]. For example, Bogart et al. [66] found that discrimination on the basis of HIV status or sexual orientation had stronger impacts on depression outcomes than racial discrimination for HIV-positive Black men. In other literature, however, racial discrimination appears to have a stronger health impact than other forms of discrimination [35, 38]. In addition, some studies have found differential strength of associations between particular forms of discrimination and particular health outcomes [40]. This suggests that the context of discrimination is important, as well as the specific health outcomes being examined, and the responses to discrimination involved [66]. While the impact of non-racial forms of discrimination appeared to be as strong as for racial forms of discrimination in our findings, it is important to remember that racial discrimination was by far the most commonly reported form of discrimination, aligning with our conceptual approach by which we understand racism to be a primary structuring phenomenon in Aotearoa/New Zealand. Racial discrimination is more common for Māori, Asian and Pacific ethnic groups, as is report of any experience of discrimination. Therefore, while the health impact of discrimination may be similar for different ethnic groupings, the burden of exposure is not [29], and hence health will be affected more for those groups with the greatest exposure. As Thoma and Huebner [10] note, people from multiple minoritised groups “…often have no completely safe or stress-free social environment” (p. 2).

### Limitations

Limitations of this study include that we were not able to determine whether or not reported multiple forms of discrimination occurred at a single point in time (e.g. discriminated against on basis of ethnicity and gender in one event) or at different times over the 12-month period (e.g. discriminated against on basis of ethnicity in one event, on basis of gender in another event), which has been shown to be an important consideration in other studies [32]. This is because the way in which the GSS asked participants about their recent history of discrimination did not enable us to assess the temporal patterns of experience of discrimination. The need for measures that are better able to assess multiple discriminations has been noted [28], including measures that identify if multiple discriminations were contemporaneous, to help us better understand the pathways for discrimination and illuminate opportunities for intervention. A further limitation was that decisions about the categorisation of ethnicity in our study were restricted by the way in which data had been pre-aggregated in the CURF. Specifically, we were unable to disaggregate ethnicities within the broad ‘Other’ category with the broad ‘European’ category. Although small numbers in the ‘Other’ category mean the impact on estimates is likely to be small, being able to separate out from the broad ‘European’ grouping would have aligned more closely with our conceptual framework. As the study is cross-sectional, we are not able to draw strong conclusions about causality. This is an area where longitudinal studies would provide important information about the direction of associations and the impacts of multiple discriminations over time. The measures for both the exposure (e.g. discrimination) and outcomes (i.e. health and wellbeing) in the survey were self-reported, and are subject to the general limitations of self-report data. With regards to discrimination in particular, the measures used in this study are likely to have under-estimated exposure to discrimination for a number of reasons. General issues include, difficulty recognising subtle or ambiguous discrimination [67, 68], social acceptability of reporting discrimination [4, 68, 69], implicit vs. explicit cognition of discrimination, denial of discrimination and difficulty determining unfair treatment without knowledge of how other groups are treated [4, 67, 68]. More specifically, this study uses a two-step question to determine different types of discrimination, whereby participants are asked about any experience of unfair treatment and then asked to attribute this to a specific reason such as race/ethnicity [68, 70]. This has been shown to produce lower reports of particular types of discrimination than a one-step question [70], although it has been suggested that one-step questions may be more prone to vigilance bias [67]. Finally, the multifarious nature of racism means that there are likely settings and types of discrimination that have not been captured in the questions used here e.g. vicarious discrimination, personal attacks, online experiences [60]. Finally, we were somewhat limited in our ability to examine whether or not the negative health impacts of exposure to multiple discriminations were *additive* (i.e. each additional form or type of discrimination has an additional health impact) [10, 46, 48], *exacerbating* [10, 46] or *interactionist* [48] (i.e. where additional forms have a multiplicative effect), or ‘inuring’ [46], whereby additional forms of discrimination do not significantly increase the negative health impacts over one form [10]. The ability of quantitative methods to assess the ways in which multiple discriminations might work together to impact health has been discussed elsewhere in the literature [32, 36, 71].

## Conclusions

Our study is one of the first studies to report the patterning of experience of multiple forms of discrimination in Aotearoa/New Zealand using nationally-representative survey data, adding to the limited literature from outside of the United States and for indigenous peoples. Our findings support the evidence documenting the negative impacts of discrimination on health, and suggest that there are additional impacts from experiencing multiple forms of discrimination. In line with comparable international findings, socially-privileged ethnic groups experienced less discrimination overall and lower exposure to multiple discriminations.

It is critical that policy responses to discrimination and interventions to dismantle oppressive systems take account of the disproportionate harm to indigenous peoples and other minoritised ethnic groups from exposure to multiple discrimination. To facilitate this, it is recommended that experiences of multiple discrimination in Aotearoa/New Zealand are routinely measured and reported in national surveys and official statistics. In addition, there is a need for research that has a more specific and in-depth focus on experiences of discrimination among those who are impacted by multiple stigmatisation and marginalisation.

This study provides insights into the pathways by which social hierarchies and health inequities are (re) produced and maintained over time. There is a pressing need for research and policy that more fully accounts for the complex lives that people live in racialised and colonial societies, in order to better conceptualise and work to eliminate health inequities. If urgent goals of health equity are to be achieved, it is important that research and practice moves away from a narrow focus on social identities and engages with the structured systems of oppression that maintain privilege and disadvantage through interlocking and embedded processes and practices. In the Aotearoa/New Zealand context, this requires critical attention to colonialism as a fundamental and persistent determinant of health and wellbeing.

## Additional files

Additional file 1: **Table S1.** Patterning of racial and other forms of discrimination in last 12 months, General Social Survey 2010. **Table S2.** Patterning of racial and other forms of discrimination in last 12 months, General Social Survey 2008. **Table S3.** Discrimination by participant characteristics and health outcomes, General Social Survey 2010. **Table S4.** Discrimination by participant characteristics and health outcomes, General Social Survey 2008. (DOCX 73 kb)

Additional file 2: Figure S1. Discrimination and self-rated health, by GSS instance and pooled estimates, unadjusted, Adjusted for age, gender, educational qualification and area-based deprivation. (TIFF 543 kb)

Additional file 3: Figure S2. Discrimination and life (dis)satisfaction, by GSS instance and pooled estimates, unadjusted, Adjusted for age, gender, educational qualification and area-based deprivation. (TIFF 546 kb)

Additional file 4: Figure S3. Discrimination and SF-12 mental health score, by GSS instance and pooled estimates, unadjusted, Adjusted for age, gender, educational qualification and area-based deprivation. (TIFF 553 kb)

Additional file 5: Figure S4. Discrimination and self-rated health, by GSS instance and pooled estimates, adjusted, Adjusted for age, gender, educational qualification and area-based deprivation. (TIFF 543 kb)

Additional file 6: Figure S5. Discrimination and life (dis)satisfaction, by GSS instance and pooled estimates, adjusted, Adjusted for age, gender, educational qualification and area-based deprivation. (TIFF 545 kb)

Additional file 7: Figure S6. Discrimination and SF-12 mental health score, by GSS instance and pooled estimates, adjusted Legend: Adjusted for age, gender, educational qualification and area-based deprivation. (TIFF 553 kb)

## Abbreviations

CURF: Confidentialised unit record files
GSS: General social survey
