# Supplementary material for: Multiple forms of discrimination and relationships with health and wellbeing: findings from national cross-sectional surveys in Aotearoa/New Zealand
Source: Int J Equity Health. 2018 Feb 17;17:26. doi: 10.1186/s12939-018-0735-y (PMC5816516; doi:10.1186/s12939-018-0735-y)
Supplement: Supplementary file 1 — Table S1. Patterning of racial and other forms of discrimination in last 12 months, General Social Survey 2010. Table S2. Patterning of racial and other forms of discrimination in last 12 months, General Social Survey 2008. Table S3. Discrimination by participant characteristics and health outcomes, General Social Survey 2010. Table S4. Discrimination by participant characteristics and health outcomes, General Social Survey 2008. (DOCX 73 kb) [file 12939_2018_735_MOESM1_ESM.docx]

**Table S1: Patterning of racial and other forms of discrimination in last 12 months, General Social Survey 2010**

|  | | Racism | | | Dress/  appearance | | | | Gender | | | | Age | | | | Disability | | | | Marital status | | | | Family | | | Sexual orientation | | | | Occupation | | | | Religion | | | | Political | | | | Other | |  |
| --- | --- | --- | --- | --- | --- | --- | --- | --- | --- | --- | --- | --- | --- | --- | --- | --- | --- | --- | --- | --- | --- | --- | --- | --- | --- | --- | --- | --- | --- | --- | --- | --- | --- | --- | --- | --- | --- | --- | --- | --- | --- | --- | --- | --- | --- | --- |
|  | | % (95% CI) | | | % (95% CI) | | | | % (95% CI) | | | | % (95% CI) | | | | % (95% CI) | | | | % (95% CI) | | | | % (95% CI) | | | % (95% CI) | | | | % (95% CI) | | | | % (95% CI) | | | | % (95% CI) | | | | % (95% CI) | | n |
| *Ethnic grouping* |  | | | | |  | | | | |  | | | | |  | | | |  | | | |  | | | | | |  | | | |  | | | |  | | | |  | | | |  |
| Māori | | | 9.7 (6.9, 12.5) | | | | 5.4 (3.3, 7.4) | | | 3.4 (1.7, 5.1) | | | | 3.1 (1.5, 4.7) | | | | 1.7 (0.6, 2.9 | | | | 1.4 (0.6, 2.3) | | | | 1.6 (0.1, 3.1) | | | 0.8 (0.0, 1.7) | | | | 2.1 (0.6, 3.6) | | | | 1.2 (0.4, 1.9) | | | | 0.7 (0.1, 1.3) | | | | 2.1 (1.0, 3.2) | 947 |
| Pacific | | | | 9.2 (5.2, 13.2) | | | 5.2 (2.0, 8.4) | | | 3.7 (1.1, 6.4) | | | | 2.5 (0.4, 4.6) | | | | - | | | | 0.7 (0.0, 1.8) | | | | 1.1 (0.0, 2.6) | | | 1.3 (0.0, 3.2) | | | | 1.4 (0.0, 2.9) | | | | 2.7 (0.2, 5.1) | | | | 1.3 (0.0, 2.8) | | | | 1.3 (0.0, 3.0) | 289 |
| Asian | | | | 15.5 (12.1, 18.8) | | | 1.8 (0.4, 3.2) | | | 1.2 (0.4, 2.0) | | | | 2.2 (0.6, 3.8) | | | | 0.3 (0.0, 0.6) | | | | - | | | | 0.3 (0.0, 0.8) | | | 0.2 (0.0, 0.5) | | | | 1.2 (0.2, 2.2) | | | | 1.7 (0.3, 3.0) | | | | 0.1 (0.0, 0.3) | | | | 1.0 (0.2, 1.8) | 571 |
| Euro/Other | | | | 3.4 (2.9, 3.9) | | | 1.6 (1.2, 2.0) | | | 1.3 (0.9, 1.6) | | | | 1.6 (1.2, 2.0) | | | | 0.8 (0.6, 1.1) | | | | 0.5 (0.3, 0.7) | | | | 0.4 (0.2, 0.6) | | | 0.3 (0.1, 0.4) | | | | 1.4 (1.0, 1.8) | | | | 0.7 (0.5, 1.0) | | | | 0.4 (0.3, 0.6) | | | | 1.8 (1.3, 2.2) | 6779 |
| *Gender* | | | |  | | | |  | | | |  | | |  | | | |  | | | |  | | | |  | | | |  | | | |  | | | |  | | | |  | | |  |
| Female | | | | 5.4 (4.4, 6.4) | | | 2.0 (1.5, 2.6) | | | 2.1 (1.6, 2.6) | | | | 2.1 (1.5, 2.7) | | | | 0.7 (0.5, 1.0) | | | | 0.8 (0.5, 1.1) | | | | 1.0 (0.5, 1.5) | | | 0.3 (0.1, 0.5) | | | | 1.5 (0.9, 2.0) | | | | 1.0 (0.6, 1.3) | | | | 0.3 (0.2, 0.5) | | | | 1.8 (1.3, 2.4) | 4773 |
| Male | | | | 5.8 (4.9, 6.7) | | | 2.4 (1.8, 3.0) | | | 1.1 (0.6, 1.6) | | | | 1.6 (1.1, 2.2) | | | | 1.0 (0.6, 1.4) | | | | 0.3 (0.1, 0.5) | | | | 0.1 (0.0, 0.2) | | | 0.4 (0.1, 0.7) | | | | 1.4 (1.0, 1.9) | | | | 0.9 (0.5, 1.3) | | | | 0.6 (0.3, 0.9) | | | | 1.6 (1.1, 2.0) | 3777 |
| *Age group* | | | |  | | |  | | |  | | | |  | | | |  | | | |  | | | |  | | |  | | | |  | | | |  | | | |  | | | |  |  |
| 15–24 | | | | 6.3 (4.6, 7.9) | | | 4.3 (2.7, 5.9) | | | 2.4 (1.0, 3.8) | | | | 4.1 (2.5, 5.8) | | | | 0.9 (0.0, 1.8) | | | | 0.5 (0.0, 1.0) | | | | 0.3 (0.0, 0.6) | | | 0.9 (0.1, 1.7) | | | | 0.9 (0.3, 1.6) | | | | 1.6 (0.7, 2.5) | | | | 0.4 (0.0, 0.7) | | | | 2.5 (1.4, 3.6) | 912 |
| 25–34 | | | | 7.3 (5.6, 9.1) | | | 2.9 (1.7, 4.0) | | | 2.0 (1.1, 2.8) | | | | 1.5 (0.7, 2.4) | | | | 0.5 (0.1, 0.9) | | | | 0.5 (0.1, 0.9) | | | | 1.0 (0.0, 2.0) | | | 0.2 (0.0, 0.4) | | | | 1.1 (0.4, 1.8) | | | | 0.9 (0.3, 1.5) | | | | 0.2 (0.0, 0.5) | | | | 1.8 (0.6, 2.9) | 1146 |
| 35–44 | | | | 7.7 (6.1, 9.3) | | | 2.5 (1.6, 3.5) | | | 2.0 (1.3, 2.8) | | | | 0.8 (0.4, 1.2) | | | | 1.3 (0.6, 1.9) | | | | 1.3 (0.6, 2.0) | | | | 1.1 (0.4, 1.8) | | | 0.2 (0.0, 0.6) | | | | 2.1 (1.3, 2.9) | | | | 0.9 (0.4, 1.4) | | | | 0.7 (0.2, 1.2) | | | | 1.4 (0.8, 2.0) | 1652 |
| 45–54 | | | | 6.5 (4.7, 8.4) | | | 2.3 (1.2, 3.4) | | | 1.9 (0.9, 2.8) | | | | 1.7 (0.7, 2.7) | | | | 1.3 (0.6, 2.0) | | | | 0.4 (0.0, 0.8) | | | | 0.7 (0.0, 1.5) | | | 0.4 (0.1, 0.8) | | | | 2.7 (1.3, 4.2) | | | | 0.9 (0.4, 1.4) | | | | 0.8 (0.3, 1.2) | | | | 1.9 (1.2, 2.7) | 1502 |
| 55–64 | | | | 3.8 (2.7, 4.9) | | | 0.6 (0.2, 1.0) | | | 1.0 (0.4, 1.5) | | | | 1.9 (1.1, 2.7) | | | | 0.8 (0.3, 1.3) | | | | 0.3 (0.0, 0.8) | | | | 0.2 (0.0, 0.5) | | | 0.2 (0.0, 0.4) | | | | 1.6 (0.8, 2.4) | | | | 1.0 (0.3, 1.8) | | | | 0.2 (0.0, 0.5) | | | | 1.5 (0.8, 2.3) | 1359 |
| 65–74 | | | | 1.6 (0.8, 2.4) | | | - | | | 0.3 (0.0, 0.5) | | | | 0.7 (0.2, 1.3) | | | | 0.2 (0.0, 0.5) | | | | 0.1 (0.0, 0.2) | | | | 0.1 (0.0, 0.3) | | | - | | | | 0.3 (0.0, 0.6) | | | | 0.1 (0.0, 0.3) | | | | 0.2 (0.0, 0.5) | | | | 0.8 (0.3, 1.3) | 1033 |
| 75+ | | | | 0.7 (0.1, 1.3) | | | 0.1 (0.0, 0.2) | | | - | | | | 1.2 (0.4, 2.0) | | | | 0.4 (0.0, 0.7) | | | | 0.1 (0.0, 0.4) | | | | 0.1 (0.0, 0.2) | | | - | | | | - | | | | 0.3 (0.0, 0.6) | | | | 0.2 (0.0, 0.5) | | | | 1.0 (0.3, 1.8) | 946 |
| *Education* | | | |  | | |  | | |  | | | |  | | | |  | | | |  | | | |  | | |  | | | |  | | | |  | | | |  | | | |  |  |
| No secondary qualification | | | | 4.1 (2.9, 5.2) | | | 2.2 (1.2, 3.1) | | | 1.3 (0.5, 2.1) | | | | 1.2 (0.6, 1.8) | | | | 1.3 (0.5, 2.2) | | | | 0.5 (0.1, 0.8) | | | | 0.4 (0.0, 0.8) | | | 0.2 (0.0, 0.5) | | | | 0.8 (0.3, 1.3) | | | | 0.7 (0.2, 1.1) | | | | 0.3 (0.0, 0.7) | | | | 1.8 (1.0, 2.6) | 2077 |
| Secondary qualification | | | | 6.0 (5.2, 6.8) | | | 2.2 (1.7, 2.7) | | | 1.7 (1.3, 2.1) | | | | 2.1 (1.6, 2.5) | | | | 0.7 (0.5, 0.9) | | | | 0.6 (0.3, 0.8) | | | | 0.6 (0.3, 1.0) | | | 0.4 (0.2, 0.6) | | | | 1.6 (1.2, 2.1) | | | | 1.0 (0.7, 1.3) | | | | 0.5 (0.3, 0.6) | | | | 1.7 (1.2, 2.1) | 6459 |
| *Quintile* | | | |  | | |  | | |  | | | |  | | | |  | | | |  | | | |  | | |  | | | |  | | | |  | | | |  | | | |  |  |
| Quintile 1 | | | | 4.3 (2.9, 5.7) | | | 1.0 (0.1, 2.0) | | | 1.5 (0.7, 2.4) | | | | 1.7 (0.8, 2.6) | | | | 0.6 (0.0, 1.2) | | | | 0.2 (0.0, 0.5) | | | | 0.4 (0.0, 1.1) | | | 0.2 (0.0, 0.6) | | | | 1.7 (0.8, 2.6) | | | | 0.3 (0.1, 0.5) | | | | 0.4 (0.1, 0.8) | | | | 1.2 (0.6, 1.7) | 1518 |
| Quintile 2 | | | | 4.8 (3.4, 6.1) | | | 1.3 (0.6, 2.0) | | | 1.4 (0.7, 2.0) | | | | 1.9 (0.9, 2.8) | | | | 0.5 (0.1, 0.8) | | | | 0.4 (0.0, 0.8) | | | | 0.4 (0.1, 0.8) | | | 0.2 (0.0, 0.4) | | | | 1.4 (0.6, 2.2) | | | | 0.6 (0.1, 1.0) | | | | 0.4 (0.1, 0.7) | | | | 1.3 (0.7, 1.9) | 1784 |
| Quintile 3 | | | | 6.7 (5.2, 8.2) | | | 3.3 (1.9, 4.7) | | | 1.7 (0.9, 2.5) | | | | 1.9 (1.1, 2.8) | | | | 0.5 (0.2, 0.9) | | | | 0.5 (0.0, 1.0) | | | | 0.4 (0.0, 0.7) | | | 0.7 (0.1, 1.3) | | | | 1.8 (1.1, 2.5) | | | | 1.6 (0.8, 2.5) | | | | 0.7 (0.2, 1.3) | | | | 1.5 (0.7, 2.2) | 1874 |
| Quintile 4 | | | | 5.5 (3.9, 7.0) | | | 2.3 (1.4, 3.2) | | | 1.5 (1.0, 2.0) | | | | 2.1 (1.2, 3.1) | | | | 1.2 (0.6, 1.8) | | | | 1.1 (0.5, 1.6) | | | | 0.6 (0.2, 0.9) | | | 0.2 (0.0, 0.5) | | | | 1.1 (0.2, 2.0) | | | | 1.0 (0.5, 1.5) | | | | 0.3 (0.0, 0.6) | | | | 2.3 (1.2, 3.5) | 1898 |
| Quintile 5 | | | | 7.0 (5.5, 8.4) | | | 3.2 (2.1, 4.2) | | | 2.1 (1.2, 2.9) | | | | 1.7 (0.9, 2.5) | | | | 1.7 (0.9, 2.4) | | | | 0.6 (0.1, 1.0) | | | | 1.2 (0.2, 2.1) | | | 0.3 (0.0, 0.7) | | | | 1.2 (0.5, 1.8) | | | | 1.3 (0.6, 1.9) | | | | 0.2 (0.0, 0.4) | | | | 2.3 (1.1, 3.5) | 1449 |

**Table notes:** % are weighted, but numbers (n) are unweighted; Euro/Other is an abbreviation for the European/Other category; cells marked as “-“ are not reported due to small cell sizes and confidentiality rules.

**Table S2: Patterning of racial and other forms of discrimination in last 12 months, General Social Survey 2008**

|  | | | Racism | | Dress/  appearance | | | | Gender | | | | Age | | | Disability | | Marital status | | Family | | Sexual orientation | | | Occupation | | | | Religion | | Political | | Other |  |
| --- | --- | --- | --- | --- | --- | --- | --- | --- | --- | --- | --- | --- | --- | --- | --- | --- | --- | --- | --- | --- | --- | --- | --- | --- | --- | --- | --- | --- | --- | --- | --- | --- | --- | --- |
|  | | | % (95% CI) | | % (95% CI) | | | | % (95% CI) | | | | % (95% CI) | | | % (95% CI) | | % (95% CI) | | % (95% CI) | | % (95% CI) | | | % (95% CI) | | | | % (95% CI) | | % (95% CI) | | % (95% CI) | n |
| *Ethnic grouping* | | |  | | | |  | | | |  | | | |  | | |  | |  | | |  | | | | |  | | |  | |  |  |
| Māori | | | 10.6 (7.3, 13.9) | | 3.7 (2.1, 5.2) | | | | 2.4 (1.3, 3.4) | | | | 2.3 (1.0, 3.6) | | | 1.4 (0.3, 2.6) | | 1.2 (0.6, 1.8) | | 0.7 (0.3, 1.2) | | 0.7 (0.0, 1.6) | | | 2.7 (1.1, 4.3) | | | | 1.8 (0.1, 3.4) | | 1.1 (0.4, 1.8) | | 3.4 (2.0, 4.7) | 972 |
| Pacific | | | 8.8 (5.1, 12.6) | 3.2 (1.1, 5.3) | | | | 2.3 (0.3, 4.4) | | | | 1.4 (0.0, 2.8) | | | | 1.9 (0.3, 3.6) | | 0.7 (0.0, 1.9) | | 0.4 (0.0, 0.9) | | 0.2 (0.0, 0.7) | | | | 2.3 (0.3, 4.2) | | | 1.7 (0.2, 3.2) | | 1.0 (0.0, 2.7) | | 1.5 (0.0, 3.2) | 385 |
| Asian | 21.4 (16.8, 26.0) | | | 1.7 (0.6, 2.7) | | | | 1.0 (0.2, 1.7) | | | | - | | | | 0.3 (0.0, 1.0) | | 0.3 (0.0, 0.8) | | - | | 0.6 (0.0, 1.7) | | | | 1.3 (0.1, 2.5) | | | 0.6 (0.0, 1.2) | | 0.1 (0.0, 0.3) | | 0.3 (0.0, 0.7) | 540 |
| Euro/Other | | | 3.0 (2.4, 3.6) | 1.5 (1.1, 1.8) | | | | 1.4 (1.0, 1.8) | | | | 1.4 (0.9, 1.8) | | | | 0.7 (0.5, 1.0) | | 0.5 (0.3, 0.8) | | 0.5 (0.3, 0.7) | | 0.2 (0.1, 0.4) | | | | 1.2 (0.8, 1.5) | | | 0.8 (0.5, 1.1) | | 0.5 (0.2, 0.7) | | 1.4 (1.1, 1.8) | 6865 |
| *Gender* | | |  | | |  | | | |  | | | |  | | |  | |  | |  | | |  | | |  | | |  | |  | |  |
| Female | | | 5.4 (4.4, 6.3) | 1.6 (1.2, 2.0) | | | | 2.0 (1.6, 2.5) | | | | 1.3 (0.9, 1.7) | | | | 0.9 (0.5, 1.3) | | 0.7 (0.4, 0.9) | | 0.6 (0.4, 0.8) | | 0.3 (0.0, 0.5) | | | | 1.3 (0.9, 1.7) | | | 0.6 (0.3, 0.9) | | 0.5 (0.2, 0.7) | | 1.1 (0.7, 1.4) | 4800 |
| Male | | | 6.4 (5.2, 7.6) | 2.1 (1.5, 2.8) | | | | 1.0 (0.6, 1.4) | | | | 1.5 (0.9, 2.1) | | | | 0.8 (0.4, 1.3) | | 0.6 (0.1, 1.0) | | 0.3 (0.1, 0.6) | | 0.3 (0.0, 0.7) | | | | 1.6 (1.0, 2.1) | | | 1.4 (0.7, 2.0) | | 0.6 (0.3, 0.9) | | 2.1 (1.5, 2.6) | 3921 |
| *Age group* | | |  |  | | | |  | | | |  | | | |  | |  | |  | |  | | | |  | | |  | |  | |  |  |
| 15–24 | 9.0 (6.7, 11.3) | | | 3.8 (2.4, 5.3) | | | | 1.8 (0.9, 2.8) | | | | 2.4 (1.1, 3.7) | | | | 1.0 (0.3, 1.8) | | 0.6 (0.0, 1.2) | | 0.6 (0.0, 1.2) | | 0.9 (0.1, 1.7) | | | | 1.6 (0.7, 2.4) | | | 2.1 (0.7, 3.6) | | 0.9 (0.2, 1.7) | | 1.9 (0.9, 2.9) | 958 |
| 25–34 | | | 7.3 (5.6, 9.0) | 2.0 (1.2, 2.7) | | | | 2.3 (1.4, 3.1) | | | | 1.9 (0.8, 3.0) | | | | 0.5 (0.1, 0.8) | | 0.9 (0.3, 1.4) | | 0.8 (0.3, 1.3) | | 0.4 (0.0, 1.1) | | | | 1.6 (0.8, 2.3) | | | 0.9 (0.4, 1.5) | | 0.5 (0.2, 0.9) | | 2.1 (1.0, 3.1) | 1277 |
| 35–44 | | | 6.2 (4.8, 7.5) | 2.0 (1.3, 2.7) | | | | 1.6 (0.7, 2.5) | | | | 0.5 (0.2, 0.8) | | | | 0.7 (0.2, 1.2) | | 1.1 (0.2, 1.9) | | 0.6 (0.3, 1.0) | | 0.1 (0.0, 0.2) | | | | 1.8 (1.0, 2.5) | | | 0.7 (0.3, 1.1) | | 0.4 (0.1, 0.7) | | 1.5 (0.9, 2.1) | 1690 |
| 45–54 | | | 6.4 (4.4, 8.4) | 1.6 (0.8, 2.4) | | | | 1.9 (1.0, 2.8) | | | | 1.3 (0.6, 2.0) | | | | 1.7 (0.7, 2.7) | | 0.6 (0.0, 1.2) | | 0.4 (0.1, 0.7) | | 0.3 (0.0, 0.6) | | | | 1.9 (0.8, 3.0) | | | 1.1 (0.3, 1.9) | | 0.8 (0.0, 1.5) | | 1.7 (1.0, 2.5) | 1547 |
| 55–64 | | | 4.1 (2.6, 5.5) | 0.9 (0.3, 1.4) | | | | 1.2 (0.6, 1.9) | | | | 1.4 (0.6, 2.2) | | | | 1.0 (0.4, 1.6) | | 0.4 (0.1, 0.7) | | 0.2 (0.0, 0.5) | | - | | | | 1.3 (0.4, 2.3) | | | 0.6 (0.1, 1.1) | | 0.3 (0.1, 0.6) | | 1.3 (0.6, 1.9) | 1317 |
| 65–74 | | | 1.7 (0.8, 2.7) | 0.5 (0.0, 1.2) | | | | 0.3 (0.0, 0.6) | | | | 1.1 (0.3, 1.8) | | | | 0.2 (0.0, 0.4) | | 0.1 (0.0, 0.2) | | - | | 0.1 (0.0, 0.2) | | | | 0.2 (0.0, 0.6) | | | 0.1 (0.0, 0.2) | | 0.1 (0.0, 0.3) | | 1.2 (0.4, 2.0) | 1021 |
| 75+ | | | 0.5 (0.0, 1.0) | 0.1 (0.0, 0.3) | | | | - | | | | 0.3 (0.0, 0.6) | | | | 0.1 (0.0, 0.3) | | - | | 0.0 (0.0, 0.1) | | - | | | | 0.1 (0.0, 0.4) | | |  | | 0.1 (0.0, 0.2) | | 0.2 (0.0, 0.6) | 911 |
| *Education* | | |  |  | | | |  | | | |  | | | |  | |  | |  | |  | | | |  | | |  | |  | |  |  |
| No secondary qualification | | | 4.3 (3.1, 5.5) | 2.0 (1.4, 2.7) | | | | 1.1 (0.4, 1.9) | | | | 0.8 (0.4, 1.2) | | | | 1.2 (0.6, 1.9) | | 0.8 (0.2, 1.4) | | 0.4 (0.2, 0.7) | | 0.2 (0.0, 0.5) | | | | 1.0 (0.6, 1.4) | | | 1.0 (0.1, 2.0) | | 0.2 (0.1, 0.3) | | 1.5 (0.8, 2.2) | 2296 |
| Secondary qualification | | | 6.4 (5.4, 7.3) | 1.8 (1.4, 2.2) | | | | 1.7 (1.3, 2.0) | | | | 1.5 (1.1, 2.0) | | | | 0.8 (0.4, 1.1) | | 0.6 (0.3, 0.8) | | 0.5 (0.3, 0.7) | | 0.3 (0.1, 0.6) | | | | 1.5 (1.1, 2.0) | | | 0.9 (0.6, 1.3) | | 0.6 (0.4, 0.9) | | 1.6 (1.2, 1.9) | 6405 |
| *Quintile* | | |  |  | | | |  | | | |  | | | |  | |  | |  | |  | | | |  | | |  | |  | |  |  |
| Quintile 1 | | | 3.7 (2.6, 4.8) | 1.1 (0.3, 1.9) | | | | 1.0 (0.5, 1.5) | | | | 1.5 (0.6, 2.3) | | | | 0.4 (0.0, 0.7) | | 0.3 (0.1, 0.6) | | 0.3 (0.1, 0.6) | | 0.0 (0.0, 0.1) | | | | 1.7 (0.8, 2.5) | | | 0.4 (0.0, 0.8) | | 0.2 (0.0, 0.3) | | 1.3 (0.5, 2.1) | 1561 |
| Quintile 2 | | | 5.7 (4.3, 7.1) | 1.5 (0.8, 2.1) | | | | 1.3 (0.8, 1.8) | | | | 1.2 (0.4, 2.0) | | | | 1.2 (0.4, 2.0) | | 0.2 (0.0, 0.3) | | 0.3 (0.0, 0.6) | | 0.2 (0.0, 0.4) | | | | 0.7 (0.3, 1.1) | | | 1.8 (0.6, 2.9) | | 0.6 (0.1, 1.1) | | 1.9 (1.1, 2.6) | 1830 |
| Quintile 3 | | | 5.4 (3.8, 7.0) | 1.5 (0.8, 2.1) | | | | 1.9 (0.8, 2.9) | | | | 0.8 (0.4, 1.2) | | | | 0.4 (0.1, 0.8) | | 0.6 (0.0, 1.2) | | 0.5 (0.1, 0.9) | | 0.2 (0.0, 0.4) | | | | 1.1 (0.5, 1.7) | | | 0.3 (0.0, 0.6) | | 0.5 (0.0, 1.1) | | 1.1 (0.6, 1.5) | 1890 |
| Quintile 4 | | 8.4 (6.3, 10.4) | | 2.8 (1.7, 3.9) | | | | 2.0 (1.2, 2.8) | | | | 1.9 (1.0, 2.9) | | | | 1.0 (0.5, 1.6) | | 1.2 (0.5, 1.8) | | 0.7 (0.2, 1.3) | | 0.6 (0.0, 1.3) | | | | 1.7 (1.0, 2.5) | | | 1.4 (0.6, 2.1) | | 1.0 (0.3, 1.6) | | 1.6 (0.8, 2.3) | 1924 |
| Quintile 5 | | | 6.6 (4.6, 8.7) | 2.7 (1.7, 3.7) | | | | 1.6 (0.9, 2.3) | | | | 1.4 (0.7, 2.2) | | | | 2.2 (1.1, 3.3) | | 1.0 (0.3, 1.7) | | 0.5 (0.1, 0.8) | | 0.6 (0.0, 1.2) | | | | 1.9 (0.8, 3.0) | | | 1.0 (0.2, 1.7) | | 0.4 (0.0, 0.8) | | 2.1 (1.3, 2.9) | 1488 |

**Table notes:** % are weighted, but numbers (n) are unweighted; Euro/Other is an abbreviation for the European/Other category; cells marked as “-“ are not reported due to small cell sizes and confidentiality rules.

**Table S3: Discrimination by participant characteristics and health outcomes, General Social Survey 2010**

|  | **Total** | **Racism only (R1)** | | **Racism plus one (R2)** | | **Racism 3+ (R3+)** | | **No R, one other (D1)** | | **No R, 2 others (D2)** | | **No R, 3 + (D3+)** | | **None** | |
| --- | --- | --- | --- | --- | --- | --- | --- | --- | --- | --- | --- | --- | --- | --- | --- |
|  | **n** | **n** | **% (95% CI)** | **n** | **% (95% CI)** | **n** | **% (95% CI)** | **n** | **% (95% CI)** | **n** | **% (95% CI)** | **n** | **% (95% CI)** | **n** | **% (95% CI)** |
| Total (n) | 8550 | 232 | 3.0 (2.5, 3.5) | 102 | 1.3 (1.0, 1.6) | 108 | 1.3 (1.0, 1.6) | 282 | 3.2 (2.7, 3.7) | 75 | 1.0 (0.7, 1.3) | 57 | 0.6 (0.4, 0.7) | 7694 | 89.6 (88.9, 90.3) |
| *Ethnicity* |  |  |  |  |  |  |  |  |  |  |  |  |  |  |  |
| Māori | 947 | 36 | 3.5 (1.8, 5.2) | 30 | 2.8 (1.6, 4.1) | 29 | 3.4 (1.6, 5.2) | 40 | 3.6 (2.3, 4.9) | 8 | 1.3 (0.2, 2.3) | 8 | 0.7 (0.2, 1.1) | 796 | 84.7 (81.1, 88.4) |
| Pacific | 289 | 15 | 4.6 (1.7, 7.6) | 4 | 1.5 (0.0, 3.1) | 13 | 3.1 (0.8, 5.4) | 8 | 2.9 (0.8, 5.1) | 2 | 1.0 (0.0, 2.9) | 1 | 0.7 (0.0, 2,2) | 246 | 86.1 (81.2, 91.0) |
| Asian | 571 | 66 | 10.1 (7.2, 13.0) | 15 | 2.6 (0.9, 4.4) | 17 | 2.7 (2.2, 4.2) | 8 | 1.1 (0.2, 1.9) | 1 | 0.1 (0.0, 0.3) |  | - | 464 | 83.4 (79.9, 86.8) |
| European/Other | 6779 | 117 | 1.9 (1.4, 2.3) | 54 | 0.9 (0.6, 1.1) | 53 | 0.7 (0.4, 0.9) | 227 | 3.5 (2.8, 4.1) | 64 | 1.1 (0.7, 1.4) | 48 | 0.6 (0.4, 0.8) | 6216 | 91.4 (90.7, 92.42) |
| *Age group* |  |  |  |  |  |  |  |  |  |  |  |  |  |  |  |
| 15-24 | 912 | 30 | 3.1 (1.9, 4.3) | 13 | 1.3 (0.5, 2.2) | 19 | 1.9 (0.9, 2.8) | 44 | 5.1 (3.5, 6.7) | 17 | 2.0 (0.9, 3.1) | 9 | 0.9 (0.2, 1.5) | 780 | 85.7 (83.2, 88.2) |
| 25-34 | 1146 | 46 | 4.1 (2.6, 5.5) | 21 | 2.2 (1.3, 3.1) | 15 | 1.1 (0.5, 1.8) | 30 | 2.3 (1.1, 3.5) | 14 | 1.6 (0.4, 2.9) | 6 | 0.4 (0.1, 0.7) | 1014 | 88.3 (85.9, 90.8) |
| 35-44 | 1652 | 66 | 4.4 (2.9, 5.8) | 23 | 1.4 (0.7, 2.2) | 36 | 1.9 (1.2, 2.6) | 58 | 2.9 (2.0, 3.9) | 18 | 1.0 (0.4, 1.6) | 15 | 0.6 (0.2, 0.9) | 1436 | 87.8 (85.9, 89.6) |
| 45-54 | 1502 | 47 | 3.6 (2.1, 5.2) | 19 | 1.3 (0.6, 2.0) | 22 | 1.6 (0.6, 2.6) | 63 | 3.8 (2.5, 5.0) | 11 | 0.5 (0.2, 0.8) | 17 | 0.9 (0.4, 1.4) | 1323 | 88.3 (86.1, 90.6) |
| 55-64 | 1359 | 26 | 1.7 (0.9, 2.5) | 18 | 1.3 (0.6, 1.9) | 13 | 0.8 (0.2, 1.5) | 47 | 3.0 (2.0, 4.1) | 9 | 0.6 (0.1, 1.1) | 6 | 0.4 (0.0, 0.8) | 1240 | 92.2 (90.6, 93.7) |
| 65-74 | 1033 | 13 | 1.2 (0.4, 1.9) | 5 | 0.3 (0.0, 0.6) | 3 | 0.2 (0.0, 0.4) | 23 | 1.9 (1.0, 2.8) | 2 | 0.2 (0.0, 0.4) | 2 | 0.2 (0.0, 0.4) | 985 | 96.3 (95.0, 97.5) |
| 75+ | 946 | 4 | 0.4 (0.0, 0.9) | 3 | 0.3 (0.0, 0.6) | 0 | - | 17 | 2.0 (1.0, 3.0) | 4 | 0.3 (0.0, 0.6) | 2 | 0.3 (0.0, 0.6) | 916 | 96.9 (95.6, 98.1) |
| *Gender* |  |  |  |  |  |  |  |  |  |  |  |  |  |  |  |
| F | 4773 | 117 | 2.7 (2.0, 3.4) | 55 | 1.2 (0.8, 1.7) | 64 | 1.5 (1.0, 2.0) | 153 | 3.3 (2.6, 3.9) | 41 | 1.1 (0.6, 1.6) | 34 | 0.6 (0.4, 0.8) | 4309 | 89.7 (88.5, 90.8) |
| M | 3777 | 115 | 3.4 (2.6, 4.2) | 47 | 1.4 (1.0, 1.7) | 44 | 1.1 (0.7, 1.5) | 129 | 3.2 (2.5, 3.9) | 34 | 0.9 (0.5, 1.3) | 23 | 0.5 (0.3, 0.8) | 3385 | 89.5 (88.4, 90.7) |
| *Education* |  |  |  |  |  |  |  |  |  |  |  |  |  |  |  |
| No secondary qualification | 2077 | 35 | 2.1 (1.2, 2.9) | 20 | 1.0 (0.0, 1.6) | 15 | 0.9 (0.3, 1.5) | 59 | 3.3 (2.0, 4.5) | 18 | 1.0 (0.4, 1.6) | 6 | 0.5 (0.0, 0.9) | 1924 | 91.2 (89.6, 92.9) |
| At least secondary qualification | 6459 | 193 | 3.3 (2.7, 3.9) | 47 | 1.4 (1.0, 1.7) | 92 | 1.4 (1.0, 1.8) | 222 | 3.2 (2.6, 3.8) | 57 | 1.0 (0.6, 1.4) | 51 | 0.6 (0.4, 0.8) | 5762 | 89.2 (88.4, 90.0) |
| *NZ Dep Quintiles* |  |  |  |  |  |  |  |  |  |  |  |  |  |  |  |
| 1 | 1518 | 36 | 2.7 (1.8, 3.7) | 12 | 0.8 (0.3, 1.3) | 9 | 0.7 (0.0, 1.5) | 45 | 3.1 (2.0, 4.3) | 9 | 0.6 (0.2, 1.1) | 10 | 0.5 (0.2, 0.8) | 1397 | 91.4 (89.7, 93.1) |
| 2 | 1784 | 42 | 2.5 (1.6, 3.4) | 18 | 1.4 (0.5, 2.3) | 12 | 0.8 (0.2, 1.4) | 54 | 3.1 (2.1, 4.1) | 16 | 1.1 (0.4, 1.8) | 7 | 0.2 (0.0, 0.4) | 1635 | 90.9 (89.5, 92.3) |
| 3 | 1874 | 59 | 4.1 (2.9, 5.3) | 24 | 1.0 (0.5, 1.5) | 27 | 1.7 (0.9, 2.4) | 59 | 3.0 (1.9, 4.1) | 17 | 1.0 (0.3, 1.7) | 15 | 0.9 (0.3, 1.5) | 1673 | 88.4 (86.6, 90.3) |
| 4 | 1898 | 46 | 2.6 (1.5, 3.8) | 25 | 1.6 (0.9, 2.4) | 27 | 1.2 (0.6, 1.7) | 70 | 3.7 (2.4, 5.1) | 20 | 1.1 (0.5, 1.8) | 14 | 0.7 (0.3, 1.1) | 1696 | 89.0 (86.9, 91.0) |
| 5 | 1449 | 49 | 3.1 (2.0, 4.2) | 23 | 1.8 (1.0, 2.6) | 33 | 2.1 (1.2, 3.0) | 53 | 3.2 (2.1, 4.3) | 13 | 1.2 (0.2, 2.3) | 11 | 0.5 (0.1, 0.9) | 1267 | 88.0 (86.0, 90.0) |
| *Self-rated health* |  |  |  |  |  |  |  |  |  |  |  |  |  |  |  |
| Poor/fair | 1287 | 32 | 4.1 (1.9, 6.3) | 23 | 2.2 (1.0, 3.4) | 23 | 1.6 (0.6, 2.6) | 65 | 4.1 (2.8, 5.4) | 24 | 2.2 (0.9, 3.4) | 15 | 1.3 (0.6, 2.1) | 1105 | 84.5 (81.5, 87.5) |
| Excellent/very good/good | 7260 | 200 | 2.9 (2.4, 3.3) | 79 | 1.2 (0.9, 1.5) | 85 | 1.2 (0.9, 1.6) | 217 | 3.1 (2.5, 3.7) | 51 | 0.8 (0.5, 1.1) | 42 | 0.5 (0.3, 0.6) | 6586 | 90.3 (89.5, 91.1) |
| *Life satisfaction* |  |  |  |  |  |  |  |  |  |  |  |  |  |  |  |
| [Very] dissatisfied | 617 | 24 | 3.9 (2.0, 5.8) | 14 | 2.8 (0.9, 4.6) | 19 | 2.4 (0.9, 1.6) | 47 | 7.5 (4.9, 10.0) | 17 | 2.0 (1.0, 3.1) | 15 | 2.5 (1.0, 4.0) | 481 | 78.9 (74.5, 83.4) |
| [Very] satisfied/ No feeling either way | 7926 | 208 | 3.0 (2.4, 3.5) | 88 | 1.2 (0.9, 1.5) | 89 | 1.2 (0.9, 1.5) | 232 | 2.9 (2.4, 3.4) | 58 | 0.9 (0.6, 1.2) | 42 | 0.4 (0.3, 0.6) | 7209 | 90.3 (89.6, 91.1) |
| *SF-12 Mental Health* |  |  |  |  |  |  |  |  |  |  |  |  |  |  |  |
| Mean (95% CI) | 8496 | 48.7 | (46.8, 50.6) | 43 | (39.8, 47.6) | 45.4 | (42.6, 48.1) | 46.2 | (44.3, 48.2_ | 48.3 | (45.6, 51.0) | 38.9 | (32.9, 44.8) | 50.4 | (50.1, 50.8) |

**Table notes:** % are weighted, but numbers (n) are unweighted

**Table S4: Discrimination by participant characteristics and health outcomes, General Social Survey 2008**

|  | **Total** | **Racism only (R1)** | | **Racism plus one (R2)** | | **Racism 3+ (R3+)** | | **No R, one other (D1)** | | **No R, 2 others (D2)** | | **No R, 3 + (D3+)** | | **None** | | |
| --- | --- | --- | --- | --- | --- | --- | --- | --- | --- | --- | --- | --- | --- | --- | --- | --- |
|  | **n** | **n** | **% (95% CI)** | **n** | **% (95% CI)** | **n** | **% (95% CI)** | **n** | **% (95% CI)** | **n** | **% (95% CI)** | **n** | **% (95% CI)** | **n** | **% (95% CI)** | |
| Total (n) | 8721 | 241 | 3.3 (2.7, 3.9) | 109 | 1.3 (1.0, 1.7) | 97 | 1.3 (0.9, 1.6) | 242 | 2.8 (2.4, 3.3) | 78 | 0.9 (0.6, 1.2) | 50 | 0.4 (0.3, 0.5) | 7904 | 90.0 (89.0, 90.9) | |
| *Ethnicity* |  |  |  |  |  |  |  |  |  |  |  |  |  |  |  | |
| Māori | 972 | 37 | 3.4 (1.9, 5.0) | 33 | 3.6 (1.8, 5.4) | 34 | 3.5 (2.0, 5.1) | 39 | 4.7 (2.4, 6.9) | 9 | 0.9 (0.2, 1.6) | 5 | 0.2 (0.0, 0.4) | 815 | 83.7 (80.3, 87.1) | |
| Pacific | 385 | 17 | 3.8 (1.0, 6.6) | 10 | 1.8 (0.6, 3.0) | 11 | 3.3 (0.7, 5.8) | 12 | 2.5 (0.6, 4.3) | 4 | 1.2 (0.0, 2.8) | 0 |  | 331 | 87.5 (83.1, 91,9) | |
| Asian | 540 | 85 | 17.4 (12.8, 22.1) | 21 | 3.0 (1.3, 4.7) | 6 | 1.0 (0.1, 1.9) | 5 | 0.8 (0.0, 1.8) | 1 | 0.1 (0.0, 0.4) | 0 |  | 422 | 77.6 (73.0, 82.2) | |
| European/Other | 6865 | 103 | 1.6 (1.2, 2.0) | 48 | 0.7 (0.5, 1.0) | 47 | 0.7 (0.4, 1.0) | 189 | 2.8 (2.3, 3.3) | 64 | 1.0 (0.6, 1.4) | 45 | 0.5 (0.3, 0.7) | 6369 | 92.6 (91.7, 93.5) | |
| *Age group* |  |  |  |  |  |  |  |  |  |  |  |  |  |  |  | |
| 15-24 | 958 | 42 | 5.3 (3.6, 7.0) | 16 | 1.7 (0.7, 2.6) | 21 | 2.0 (0.9, 3.1) | 40 | 4.5 (2.7, 6.3) | 21 | 2.0 (1.0, 3.0) | 4 | 0.2 (0.0, 0.4) | 814 | 84.3 (81.4, 87.2) | |
| 25-34 | 1277 | 51 | 4.2 (2.8, 5.7) | 23 | 2.0 (1.0, 3.0) | 15 | 1.1 (0.5, 1.8) | 48 | 3.2 (2.0, 4.3) | 14 | 1.2 (0.2, 2.2) | 14 | 0.8 (0.3, 1.3) | 1112 | 87.5 (85.2, 89.9) | |
| 35-44 | 1690 | 58 | 3.4 (2.3, 4.5) | 29 | 1.5 (1.0, 2.1) | 26 | 1.2 (0.6, 1.8) | 54 | 2.6 (1.7, 3.4) | 10 | 0.9 (0.1, 1.7) | 7 | 0.3 (0.0, 0.6) | 1506 | 90.1 (88.2, 92.0) | |
| 45-54 | 1547 | 50 | 3.4 (1.7, 5.2) | 18 | 1.0 (0.5, 1.6) | 24 | 1.9 (0.9, 3.0) | 49 | 3.3 (2.1, 4.6) | 19 | 0.7 (0.4, 1.0) | 12 | 0.4 (0.2, 0.7) | 1375 | 89.2 (86.8, 91.6) | |
| 55-64 | 1317 | 24 | 1.9 (1.0, 2.9) | 19 | 1.4 (0.5, 2.3) | 7 | 0.8 (0.1, 1.5) | 27 | 1.8 (1.0, 2.7) | 8 | 0.4 (0.1, 0.7) | 11 | 0.7 (0.2, 1.1) | 1221 | 93.0 (91.3, 94.8) | |
| 65-74 | 1021 | 14 | 1.1 (0.4, 1.8) | 1 | 0.1 (0.0, 0.4) | 4 | 0.4 (0.0, 1.0) | 15 | 1.5 (0.6, 2.4) | 6 | 0.4 (0.1, 0.7) | 2 | 0.1 (0.0, 0.3) | 979 | 96.3 (95.0, 97.6) | |
| 75+ | 911 | 2 | 0.3 (0.0, 0.6) | 3 | 0.3 (0.0, 0.6) | 0 | - | 9 | 0.8 (0.3, 1.4) | 0 | - | 0 | - | 897 | 98.6 (97.8, 99.4) | |
| *Gender* |  |  |  |  |  |  |  |  |  |  |  |  |  |  |  | |
| F | 4800 | 140 | 3.1 (2.4, 3.9) | 50 | 0.9 (0.6, 1.3) | 52 | 1.3 (0.9, 1.8) | 125 | 2.3 (1.8, 2.9) | 45 | 0.9 (0.6, 1.3) | 32 | 0.5 (0.3, 0.7) | 4356 | 90.9 (89.6, 92.2) | |
| M | 3921 | 101 | 3.4 (2.6, 4.3) | 59 | 1.7 (1.1, 2.3) | 45 | 1.2 (0.7, 1.7) | 117 | 3.4 (2.6, 4.2) | 33 | 1.0 (0.5, 1.5) | 18 | 0.3 (0.1, 0.5) | 3548 | 89.0 (87.5, 90.4) | |
| *Education* |  |  |  |  |  |  |  |  |  |  |  |  |  |  |  | |
| No secondary qualification | 2296 | 42 | 2.4 (1.5, 3.3) | 17 | 0.9 (0.4, 1.4) | 26 | 1.0 (0.5, 1.5) | 66 | 3.6 (2.4, 4.8) | 18 | 1.0 (0.3, 1.7) | 8 | 0.3 (0.1, 0.5) | 2119 | 90.8 (89.1, 92.5) | |
| At least secondary qualification | 6405 | 198 | 3.6 (2.9, 4.3) | 92 | 1.4 (1.1, 1.8) | 71 | 1.3 (0.9, 1.8) | 175 | 2.6 (2.1, 3.1) | 60 | 0.9 (0.6, 1.3) | 42 | 0.4 (0.3, 0.6) | 5767 | 89.7 (88.6, 90.8) | |
| *NZ Dep Quintiles* |  |  |  |  |  |  |  |  |  |  |  |  |  |  |  | |
| 1 | 1561 | 28 | 2.1 (1.3, 2.8) | 11 | 0.9 (0.3, 1.6) | 9 | 0.7 (0.2, 1.2) | 40 | 2.7 (1.7, 3.6) | 9 | 0.8 (0.0, 1.5) | 10 | 0.5 (0.1, 0.9) | 1454 | 92.4 (90.7, 94.0) | |
| 2 | 1830 | 51 | 3.6 (2.4, 4.8) | 22 | 1.1 (0.6, 1.6) | 17 | 0.9 (0.4, 1.4) | 49 | 3.4 (2.0, 4.9) | 14 | 0.7 (0.3, 1.1) | 9 | 0.4 (0.1, 0.8) | 1668 | 89.9 (87.8, 91.7) | |
| 3 | 1890 | 51 | 3.8 (2.3, 5.4) | 14 | 0.6 (0.2, 0.9) | 14 | 1.0 (0.3, 1.7) | 44 | 2.3 (1.5, 3.1) | 16 | 1.0 (0.3, 1.8) | 8 | 0.2 (0.1, 0.4) | 1743 | 91.0 (89.0, 93.1) | |
| 4 | 1924 | 69 | 4.5 (2.9, 6.1) | 25 | 1.8 (1.0, 2.6) | 32 | 2.1 (1.1, 3.1) | 53 | 2.4 (1.6, 3.2) | 19 | 0.8 (0.3, 1.2) | 17 | 0.6 (0.3, 0.9) | 1709 | 87.9 (85.8, 90.0) | |
| 5 | 1488 | 41 | 2.6 (1.5, 3.6) | 37 | 2.3 (1.4, 3.3) | 25 | 1.7 (0.8, 2.7) | 54 | 3.4 (2.3, 4.5) | 20 | 1.5 (0.7, 2.4) | 6 | 0.2 (0.0, 0.4) | 1305 | 88.2 (85.8, 90.7) | |
| *Self-rated health* |  |  |  |  |  |  |  |  |  |  |  |  |  |  |  | |
| Poor/fair | 1286 | 38 | 3.9 (2.1, 5.7) | 25 | 2.1 (1.2, 3.0) | 23 | 2.1 (0.9, 3.3) | 66 | 5.7 (4.0, 7.4) | 23 | 1.9 (0.8, 3.1) | 12 | 0.7 (0.3, 1.1) | 1099 | 83.6 (80.3, 86.8) | |
| Excellent/very good/good | 7435 | 203 | 3.2 (2.6, 3.8) | 84 | 1.2 (0.8, 1.6) | 74 | 1.1 (0.8, 1.4) | 176 | 2.4 (1.9, 2.9) | 38 | 0.4 (0.2, 0.5) | 38 | 0.4 (0.2, 0.5) | 6805 | 90.9 (90.0, 91.9) | |
| *Life satisfaction* |  |  |  |  |  |  |  |  |  |  |  |  |  |  |  | |
| [Very] dissatisfied | 660 | 31 | 4.9 (2.9, 6.9) | 13 | 2.6 (0.8, 4.3) | 20 | 3.0 (1.4, 4.7) | 43 | 5.9 (3.7, 8.0) | 10 | 1.0 (0.3, 1.7) | 10 | 1.0 (0.3, 1.7) | 521 | 78.8 (74.8, 82,9) | |
| [Very] satisfied/ No feeling either way | 8050 | 209 | 3.2 (2.6, 3.7) | 95 | 1.2 (0.9, 1.6) | 77 | 1.1 (0.8, 1.5) | 198 | 2.6 (2.1, 3.1) | 40 | 0.4 (0.2, 0.5) | 40 | 0.4 (0.2, 0.5) | 7376 | 90.8 (89.9, 91.7) | |
| *SF-12 Mental Health* |  |  |  |  |  |  |  |  |  |  |  |  |  |  |  | |
| Mean (95% CI) | 8655 | 49.1 | (47.5, 50.6) | 48.6 | (45.8, 51.4) | 47.5 | (44.8, 50.1) | 48.4 | (46.8, 50.1) | 44.6 | (41.0, 48.2) | 42.4 | (38.1, 46.7) | 52.9 | | (52.7, 53.2) |

**Table notes:** % are weighted, but numbers (n) are unweighted
