# Supplementary figures and images for: Multiple forms of discrimination and relationships with health and wellbeing: findings from national cross-sectional surveys in Aotearoa/New Zealand
Source: Int J Equity Health. 2018 Feb 17;17:26. doi: 10.1186/s12939-018-0735-y (PMC5816516; doi:10.1186/s12939-018-0735-y)

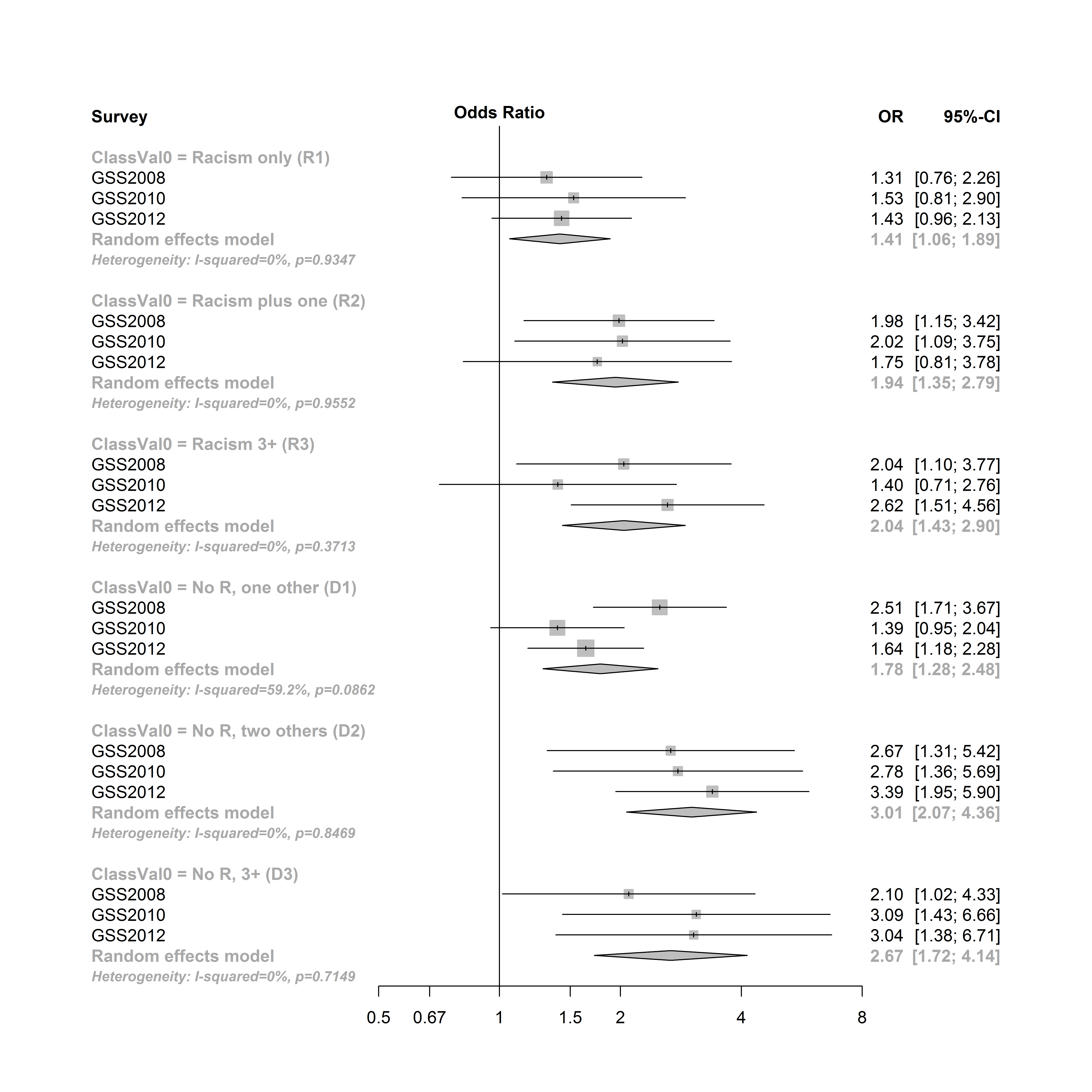

Supplement: Supplementary file 2 — Discrimination and self-rated health, by GSS instance and pooled estimates, unadjusted, Adjusted for age, gender, educational qualification and area-based deprivation. (TIFF 543 kb) [file 12939_2018_735_MOESM2_ESM.tiff]

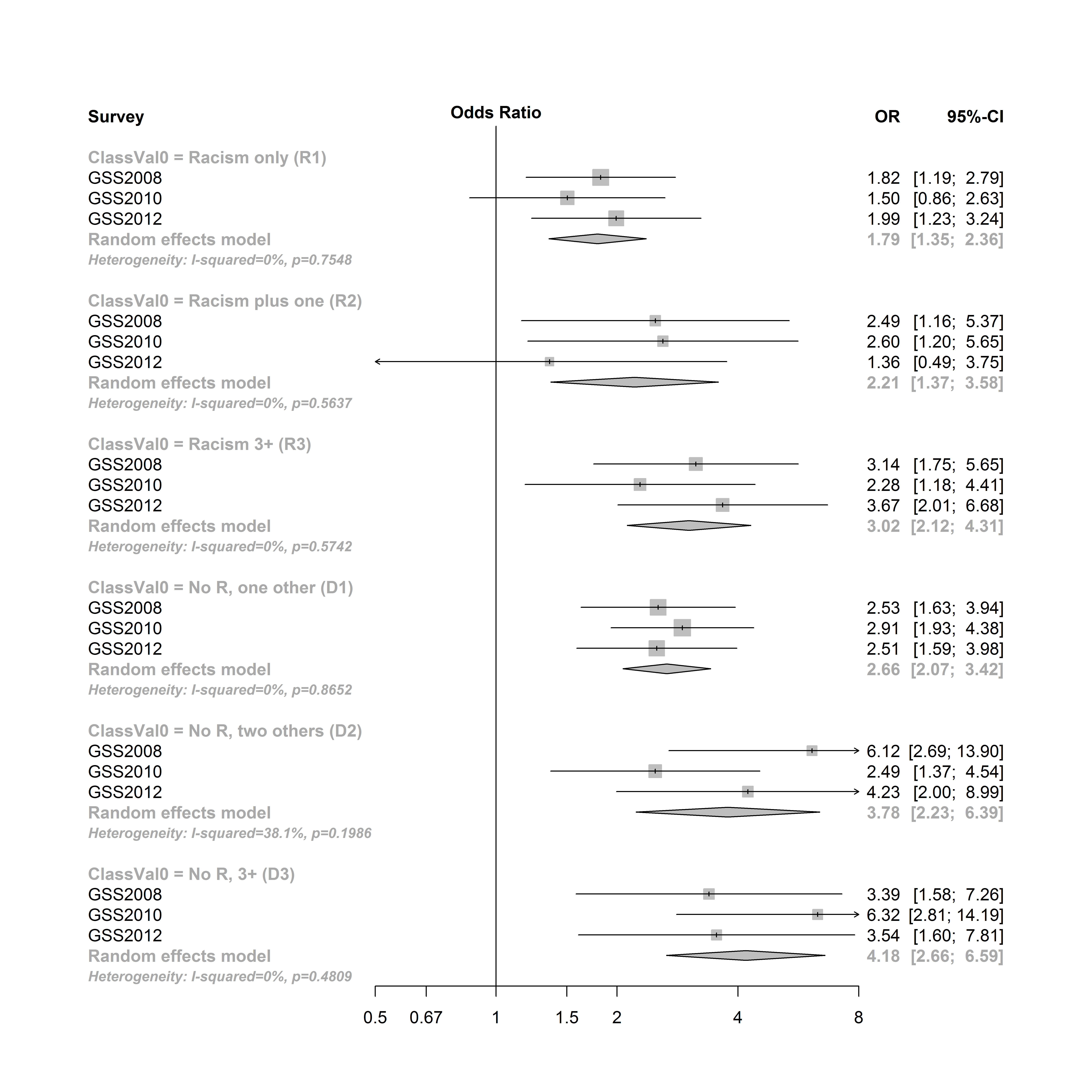

Supplement: Supplementary file 3 — Discrimination and life (dis)satisfaction, by GSS instance and pooled estimates, unadjusted, Adjusted for age, gender, educational qualification and area-based deprivation. (TIFF 546 kb) [file 12939_2018_735_MOESM3_ESM.tiff]

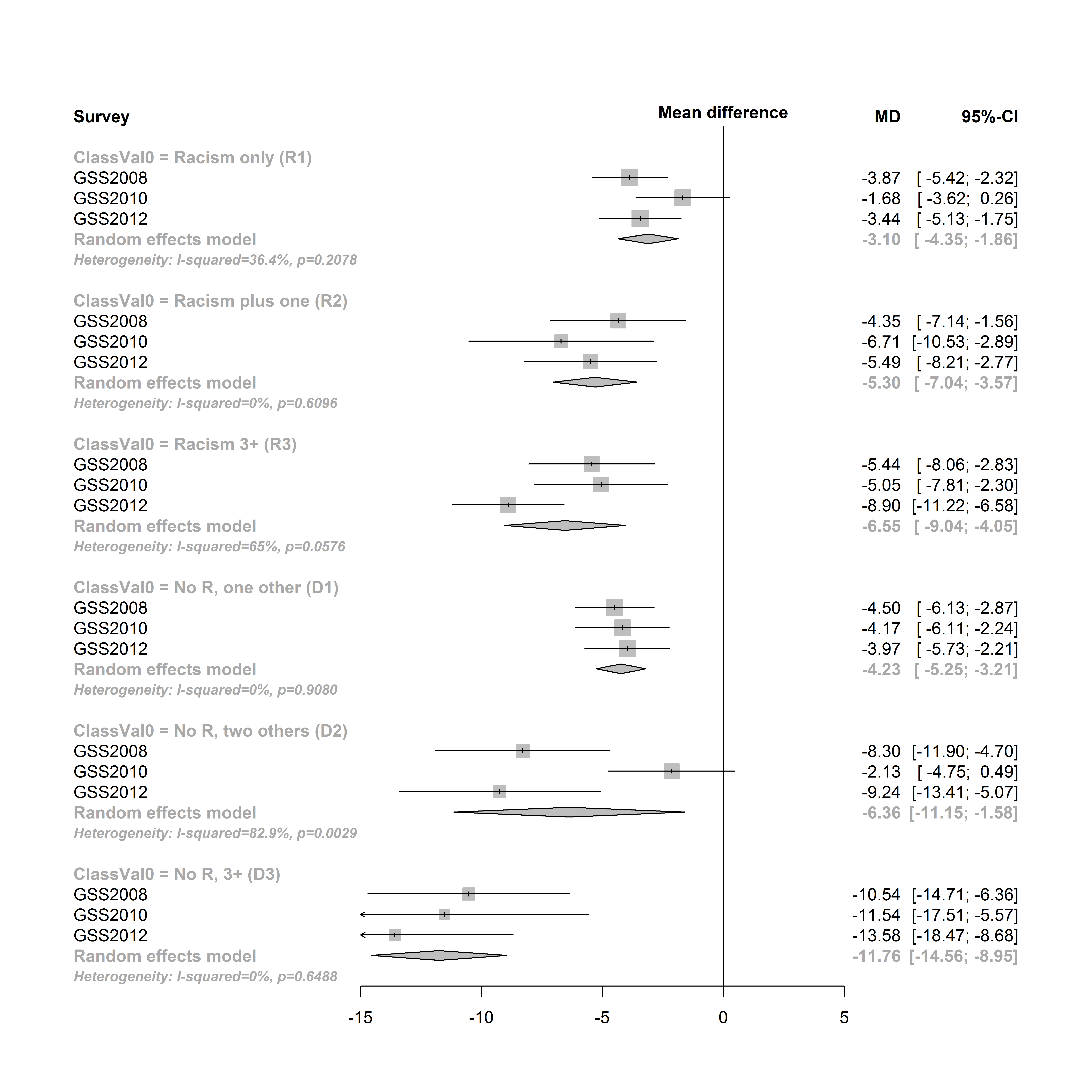

Supplement: Supplementary file 4 — Discrimination and SF-12 mental health score, by GSS instance and pooled estimates, unadjusted, Adjusted for age, gender, educational qualification and area-based deprivation. (TIFF 553 kb) [file 12939_2018_735_MOESM4_ESM.tiff]

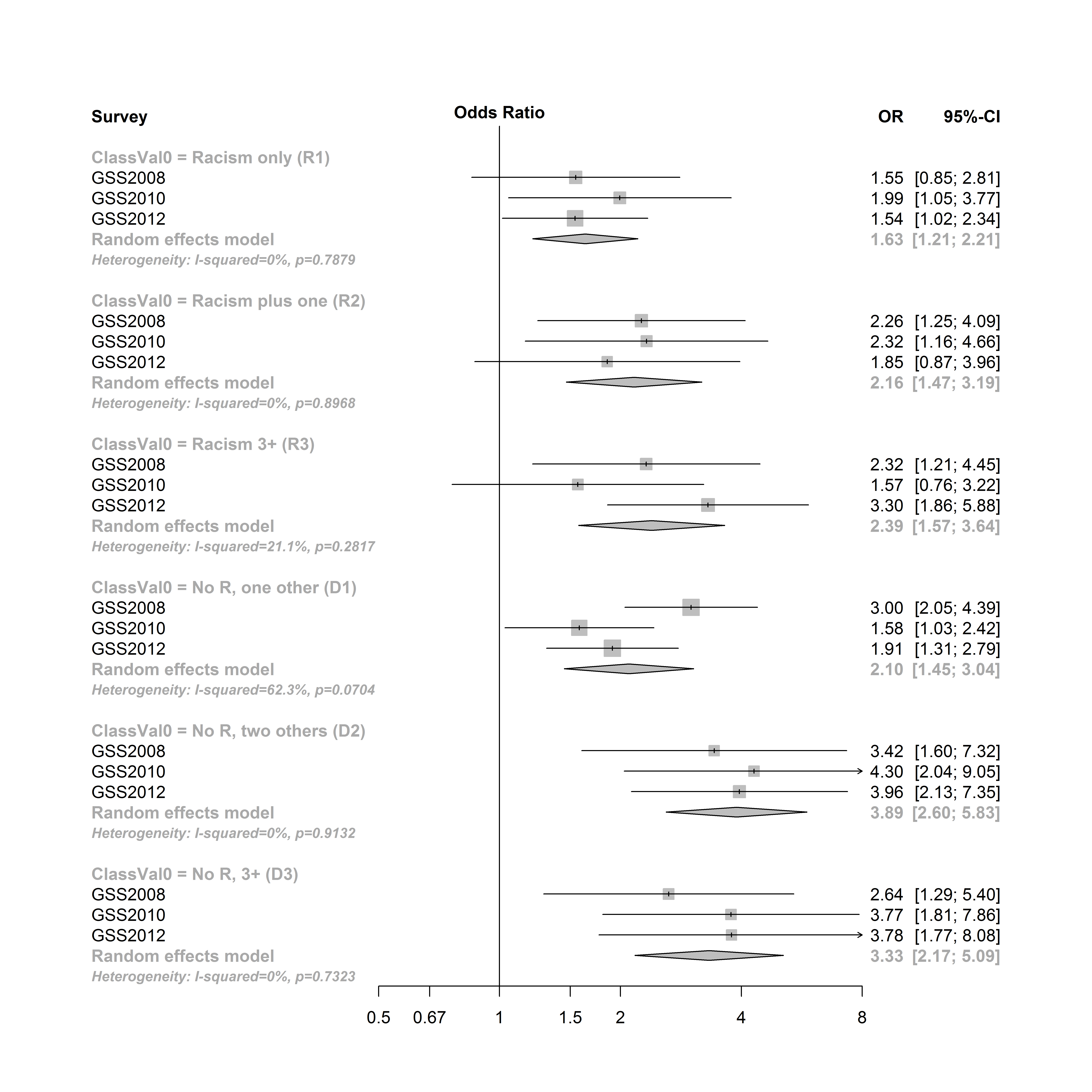

Supplement: Supplementary file 5 — Discrimination and self-rated health, by GSS instance and pooled estimates, adjusted, Adjusted for age, gender, educational qualification and area-based deprivation. (TIFF 543 kb) [file 12939_2018_735_MOESM5_ESM.tiff]

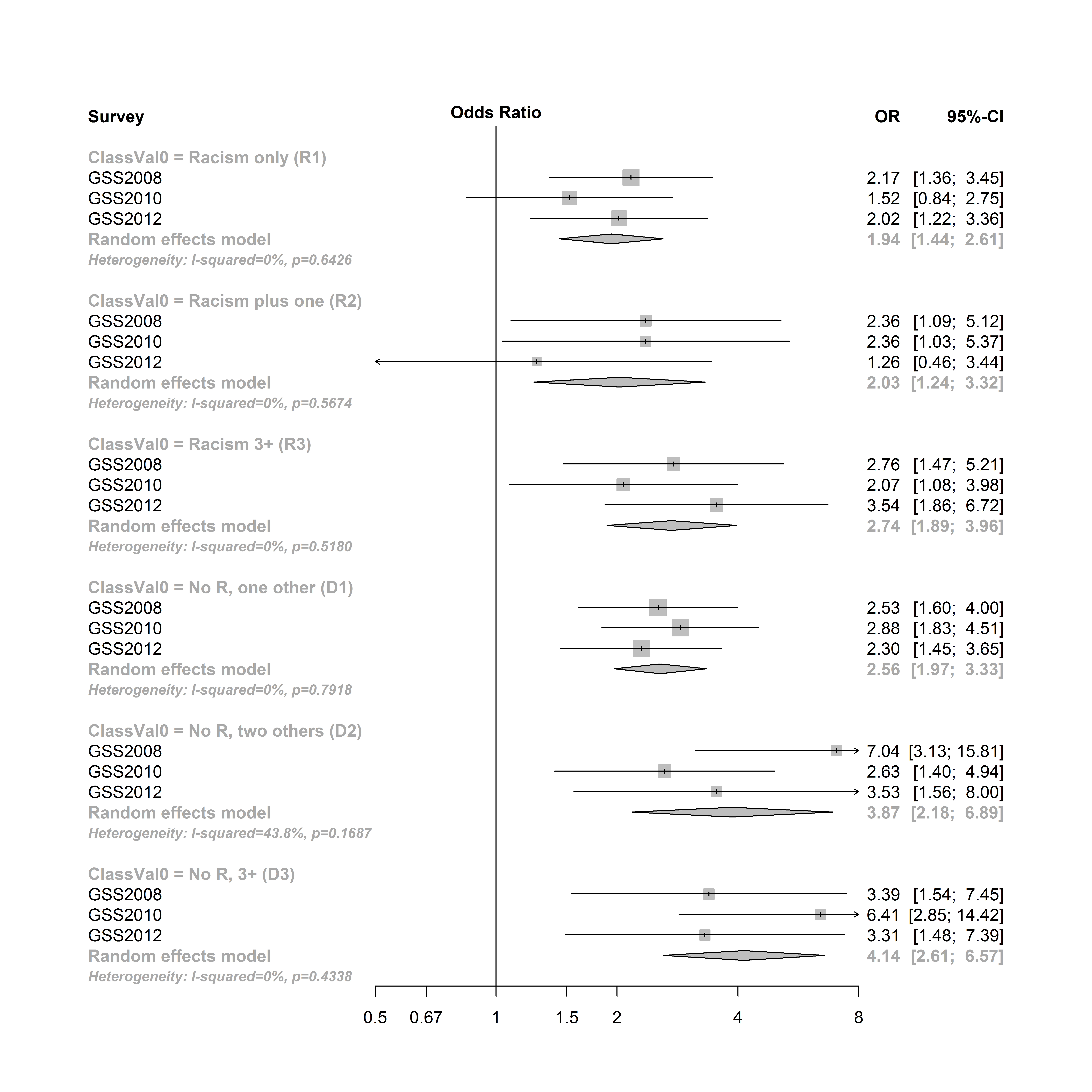

Supplement: Supplementary file 6 — Discrimination and life (dis)satisfaction, by GSS instance and pooled estimates, adjusted, Adjusted for age, gender, educational qualification and area-based deprivation. (TIFF 545 kb) [file 12939_2018_735_MOESM6_ESM.tiff]

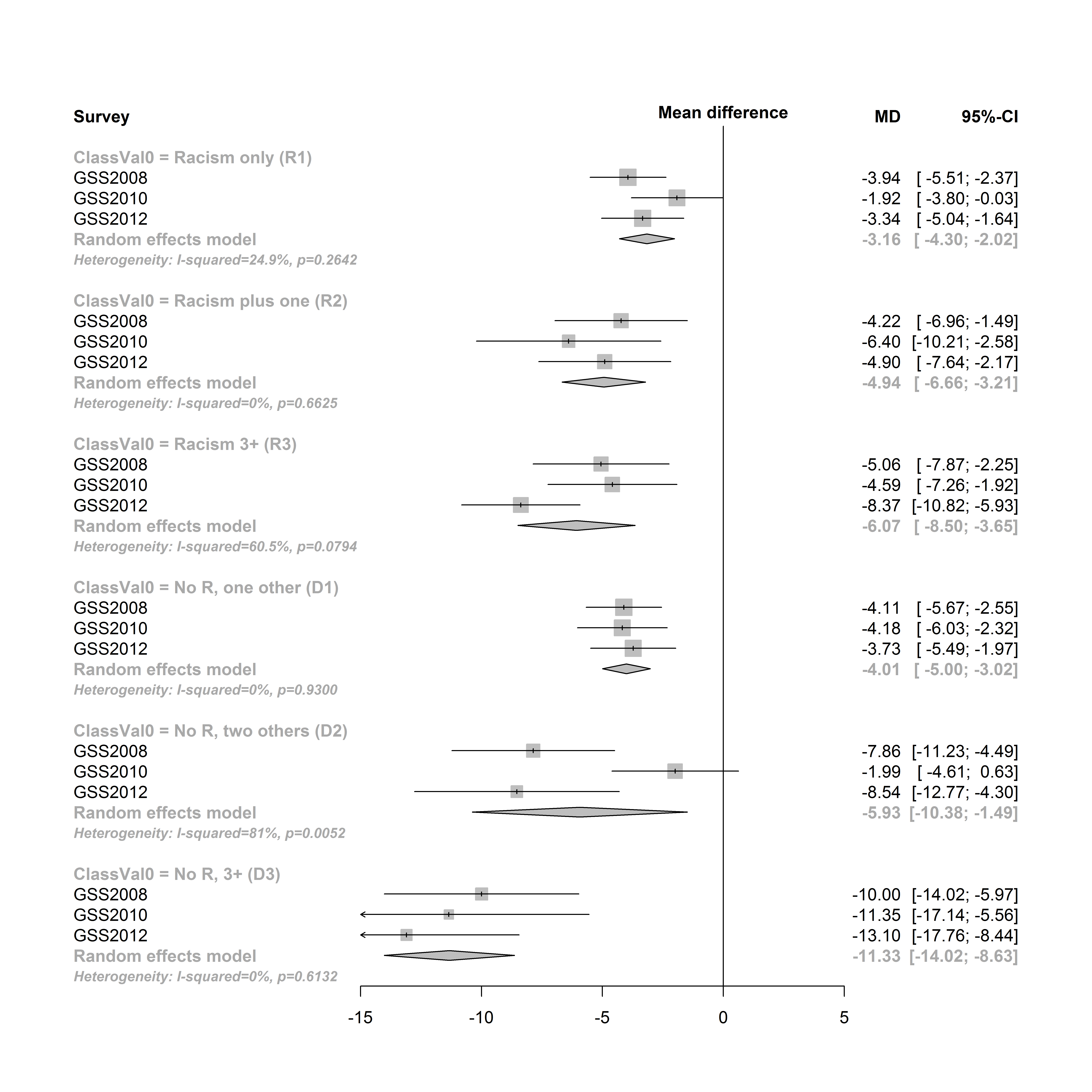

Supplement: Supplementary file 7 — Discrimination and SF-12 mental health score, by GSS instance and pooled estimates, adjusted Legend: Adjusted for age, gender, educational qualification and area-based deprivation. (TIFF 553 kb) [file 12939_2018_735_MOESM7_ESM.tiff]
